# Breaking the bottlenecks in anti-tumor angiogenic therapy: targeting vasculogenic mimicry with natural products and traditional Chinese medicine

**DOI:** 10.3389/fphar.2025.1668083

**Published:** 2025-09-12

**Authors:** Huan Liu, Yuan Zhang, Xuanyu Lv, Xueying Ding, Wenlu Liao, Weifang Sun, Yanan Zhang, Chunyan Song, Yong Tang

**Affiliations:** ^1^ College of Traditional Chinese Medicine, Changchun University of Chinese Medicine, Changchun, China; ^2^ College of Clinical Medical, Changchun University of Chinese Medicine, Changchun, China; ^3^ Department of Otorhinolaryngology Head and Neck Surgery, Changchun Central Hospital, Changchun, China

**Keywords:** vasculogenic mimicry, natural products, traditional Chinese medicine, tumor angiogenesis, extracellular matrix remodeling, epithelial-mesenchymal transition

## Abstract

Cancer, as a major public health problem threatening human health, poses significant challenges in clinical management due to its high invasiveness, metastatic potential, and therapeutic resistance. Vasculogenic mimicry (VM) is a vascular-like structure autonomously formed by highly plastic tumor cells and has been shown to be one of the significant factors influencing the progression, metastasis, and therapeutic resistance of malignant tumors. Unlike conventional anti-angiogenic therapies that primarily target endothelial cell-mediated neovascularization, VM can facilitate the transport of oxygen and nutrients in the absence of endothelial cell participation. This unique mechanism limits the efficacy of current anti-angiogenic strategies and contributes to treatment failure and tumor recurrence. Consequently, the development of novel therapeutic strategies is of paramount importance. In recent years, accumulating evidence has demonstrated that natural products (NPs) and traditional Chinese medicine (TCM), owing to their multi-component and multi-target properties, exhibit unique advantages and significant potential in inhibiting VM formation. This review systematically summarizes recent advances in the application of NPs and TCM to inhibit VM, with a focus on their key mechanisms of action in regulating cell adhesion molecules, extracellular matrix remodeling, epithelial-mesenchymal transition, cancer stemness, hypoxia adaptation, and ferroptosis. Furthermore, we summarize the anti-VM mechanisms of NPs and TCM in multiple malignant tumors such as lung cancer, liver cancer, breast cancer, and glioblastoma, and clarify their potential application prospects. These findings provide a theoretical foundation for developing VM-targeted therapies and promote the transformation and application of NPs and TCM in the field of anti-tumor VM.

## 1 Introduction

Vasculogenic mimicry (VM) refers to a process by which highly aggressive malignant tumor cells autonomously form vascular-like tubular networks in the absence of endothelial cells, thereby meeting the demand for oxygen and nutrient supply during tumor progression (Wei et al., 2021). In recent years, VM has been identified as a common phenomenon in various malignant tumors, including hepatocellular carcinoma (HCC), breast cancer, lung cancer, melanoma, and glioblastoma (Zhang et al., 2024b; Fu et al., 2021; Seftor et al., 2012). The presence of VM not only enhances tumor survival but is also closely associated with increased tumor invasiveness, metastasis, and resistance to anti-cancer therapies, particularly in highly aggressive tumors (Hsu et al., 2024; Delgado-Bellido et al., 2020; Treps et al., 2021). Unlike conventional angiogenesis, VM is characterized by the self-organization of tumor cells into three-dimensional tubular networks. These structures enable tumor cells to convey blood flow through autonomously generated vessel-like channels while also facilitating oxygen and nutrient delivery to sustain metabolic demands, thereby enabling tumors to grow and disseminate in the absence of new blood vessels (Pandiar et al., 2023; Luo et al., 2020). The primary distinction between the two lies in their mechanisms of formation and biological functions. Traditional angiogenesis establishes vascular networks through endothelial cell proliferation, migration, and interactions with the extracellular matrix (ECM), whereas VM predominantly relies on tumor cell-autonomous properties (Folberg et al., 2000).

In terms of morphological features, the structure of VM often exhibits characteristic tubular pattern. In the identification of VM, periodic acid–Schiff (PAS) staining is commonly employed to detect the presence of glycoproteins, while immunohistochemical markers CD31 and CD34 are employed to exclude the involvement of endothelial cells (Andonegui-Elguera et al., 2020; Louis et al., 2024; Wu et al., 2020). Specifically, if a tissue sample shows PAS-positive staining but is negative for both CD31 and CD34, it indicates that these structures are composed of tumor cells rather than endothelial cells, thereby supporting the presence of VM. In addition, recent studies have proposed a novel concept known as total microvessel density (TMVD), which integrates traditional microvessel density (MVD) with the presence of VM, aiming to improve the accuracy of tumor infiltration assessment (Wu et al., 2020).

Given the distinct pathogenic mechanisms between VM and classical angiogenesis, recent studies have shown that conventional anti-angiogenic agents-such as bevacizumab-which target VEGF to inhibit endothelial-dependent angiogenesis, exhibit limited efficacy against VM (Liang et al., 2020; Ribatti et al., 2021). This is primarily due to structural differences between VM and angiogenesis, as well as the activation of distinct compensatory mechanisms in tumor cells under nutrient-deficient conditions, including the upregulation of HIF-1α and epithelial-mesenchymal transition (EMT)-related transcription factors (Pandiar et al., 2023; Li et al., 2016). Moreover, the formation of VM is closely associated with tumor aggressiveness, metastatic competence, and poor prognosis, thereby increasing the complexity and difficulty of cancer treatment (Linder and Tschernig, 2016; Liu et al., 2016). Therefore, the development of more effective anti-VM strategies has become an urgent priority. Natural products (NPs) are bioactive compounds extracted from natural sources such as plants, animals, and microorganisms, characterized by rich chemical diversity (Newman and Cragg, 2020). These compounds span multiple chemical classes, primarily including alkaloids, flavonoids, terpenoids, and polyphenols. The structural diversity of NPs underpins their biological activities, as their unique chemical frameworks enable interactions with various molecular targets, thereby exhibiting a range of biological effects such as anti-tumor, anti-inflammatory, and antioxidant properties (Atanasov et al., 2015). Traditional Chinese medicine (TCM) is typically based on multi-component herbal formulations, with its efficacy deriving from the synergistic actions of multiple compounds (Li et al., 2024). The structural characteristics of TCM lie in its multi-component complexity, where interactions among different compounds enhance the overall therapeutic effect, allowing TCM to target multiple biological pathways and molecular targets simultaneously (Wang et al., 2020). Studies have demonstrated that NPs and TCM possess inherent advantages in anti-cancer therapy. Many NPs and TCM have been shown to exhibit a variety of biological activities, including anti-proliferative, anti-migratory, anti-invasive, and antioxidant effects (Yang et al., 2021; Islam et al., 2022; Sharifi-Rad et al., 2019). Their anti-tumor efficacy is achieved through multi-component, multi-target, and multi-pathway mechanisms. And they also demonstrate significant potential and therapeutic value in targeting VM. For instance, some NPs and TCMs exert anti-VM effects by regulating the expression of key VM-related molecules such as VE-cadherin, EphA2, and HIF-1α; inhibiting signaling pathways including PI3K/Akt and Wnt/β-catenin; suppressing EMT; and destroying the stemness characteristics of tumor cells (Cai et al., 2024; Chu et al., 2021; Gao et al., 2024). These findings offer new insights and therapeutic targets for the development of novel anti-cancer strategies and drugs. In view of this, this article will comprehensively summarizes the molecular mechanisms by which NPs and TCM inhibit tumor VM. It systematically organizes representative natural product monomers and herbal formulations reported in recent years with anti-VM potential, with a particular focus on their roles in cytoskeletal remodeling, EMT, tumor cell stemness, ECM remodeling, and signaling pathway regulation. By thoroughly analyzing the targets of NPs and TCM, as well as their multi-target and multi-pathway characteristics, this study aims to provide a theoretical foundation for the development of novel anti-VM therapeutics. Furthermore, it offers perspectives on future directions in this field, thereby promoting the translational application of NPs and TCM in the treatment of tumor VM.

## 2 Molecular mechanisms of tumor VM formation

### 2.1 Cell adhesion molecules and the formation of tumor vasculogenic-like structures

Cell adhesion molecules (CAMs) are transmembrane receptor proteins that mediate specific binding interactions between cells and adjacent cells or between cells and the ECM. They mainly include the cadherin family, integrin family, immunoglobulin superfamily, etc. (Kozlova and Sytnyk, 2024). Tumor VM is a form of tumor microcirculation that does not rely on endothelial cells. Its formation requires three key biological processes: dedifferentiation of tumor cells to acquire endothelial-like characteristics, ECM remodeling to establish channel structures, and connection with the host vasculature to establish a functional circulatory network (Liu et al., 2025; Zhang et al., 2019). In this process, CAMs play a central regulatory role by regulating cellular plasticity, driving the endothelial-like transdifferentiation of tumor cells, promoting the formation of VM channel networks, modulating cell polarity and migration, and maintaining the structural stability of VM channels, thereby facilitating the generation of vessel-like structures in the absence of endothelial cell involvement (Hayashi et al., 2020; Wang et al., 2015). Specifically, the primary functions of CAMs include promoting adhesion between tumor cells, facilitating adhesion between tumor cells and the ECM, maintaining the structural integrity of the channels, exerting barrier functions, and serving as signaling hubs (Liu et al., 2017; Wu et al., 2019; Liu et al., 2016).

The formation of VM first requires tumor cells to tightly adhere to one another to form tubular structures (Zhang et al., 2019). VE-Cadherin is a core molecule in endothelial cell adhesion, consisting of an extracellular domain that mediates homotypic cell–cell adhesion, and an intracellular domain that connects to β-catenin and γ-catenin, which are further anchored to the actin cytoskeleton via α-catenin, thereby maintaining the stability of the endothelial barrier (Odell et al., 2012; Guo et al., 2008; Lim et al., 2001; Abu Taha and Schnittler, 2014). VE-cadherin plays a crucial role in promoting the formation of VM channels and maintaining vascular integrity. Many studies have identified it as a representative molecule involved in tumor VM formation (Delgado-Bellido et al., 2023a). Studies have shown that tumor cells promote the replacement of endothelial cells and the formation of lumen-like structures by upregulating the expression of VE-cadherin, particularly in highly invasive and metastatic malignant tumor cells (Qin et al., 2019). The expression level of VE-cadherin is significantly positively correlated with the density of VM channels, and silencing its expression can inhibit VM formation. For example, in various tumor types such as lung cancer, esophageal cancer, and melanoma, VE-cadherin is highly expressed and enhances the VM-forming ability of tumor cells, which is also significantly associated with poor patient prognosis (Williamson et al., 2016; Qin et al., 2019; Delgado-Bellido et al., 2025; Delgado-Bellido et al., 2023a). The phosphorylation status of VE-Cadherin is a critical regulatory factor in tumor cell VM. Tumor cells enhance the phosphorylation of VE-Cadherin at the Y658 residue, which promotes the formation of a VE-Cadherin/β-catenin complex and increases the transcriptional activity of TCF-4 (Delgado-Bellido et al., 2020; Delgado-Bellido et al., 2023b). This leads to the establishment of a VE-Cadherin/β-catenin/TCF-4 signaling axis, thereby enhancing the ability of tumor cells to form vessel-like structures. Further studies have revealed that high expression of VE-Cadherin significantly upregulates the expression of PI3K, which in turn enhances the expression and activity of downstream MMP2 and MMP14. This establishes a VE-Cadherin/PI3K/MMPs signaling axis that promotes ECM degradation and remodeling, thereby creating space for the formation of tubular VM structures (Shuai et al., 2020). Elevated VE-Cadherin expression also promotes the expression of EphA2 and induces phosphorylation of FAK at Tyr397 downstream of EphA2. Activated FAK mediates phosphorylation of VE-Cadherin at the Y658 site, further facilitating the formation of a complex between phosphorylated VE-Cadherin and p120-catenin, which regulates nuclear transcription and enhances tumor cell migration and plasticity. Collectively, these events form a VE-Cadherin/EphA2/FAK/p-VE-Cadherin signaling axis that regulates VM network formation (Shuai et al., 2020). Additionally, upregulated VE-Cadherin induces EMT, characterized by downregulation of E-Cadherin and upregulation of N-Cadherin, Vimentin, Snail1, and Twist1. It also promotes the secretion of key ECM components such as Laminin, Collagen, and Fibronectin, as well as the release of angiogenic factors including VEGF, FGF-2, IL-8, IL-6, and TGF-β. These mechanisms synergistically facilitate cytoskeletal rearrangement, cell migration, invasion, and the stability of VM channels (Shuai et al., 2020; Delgado-Bellido et al., 2017).

**FIGURE 1:**
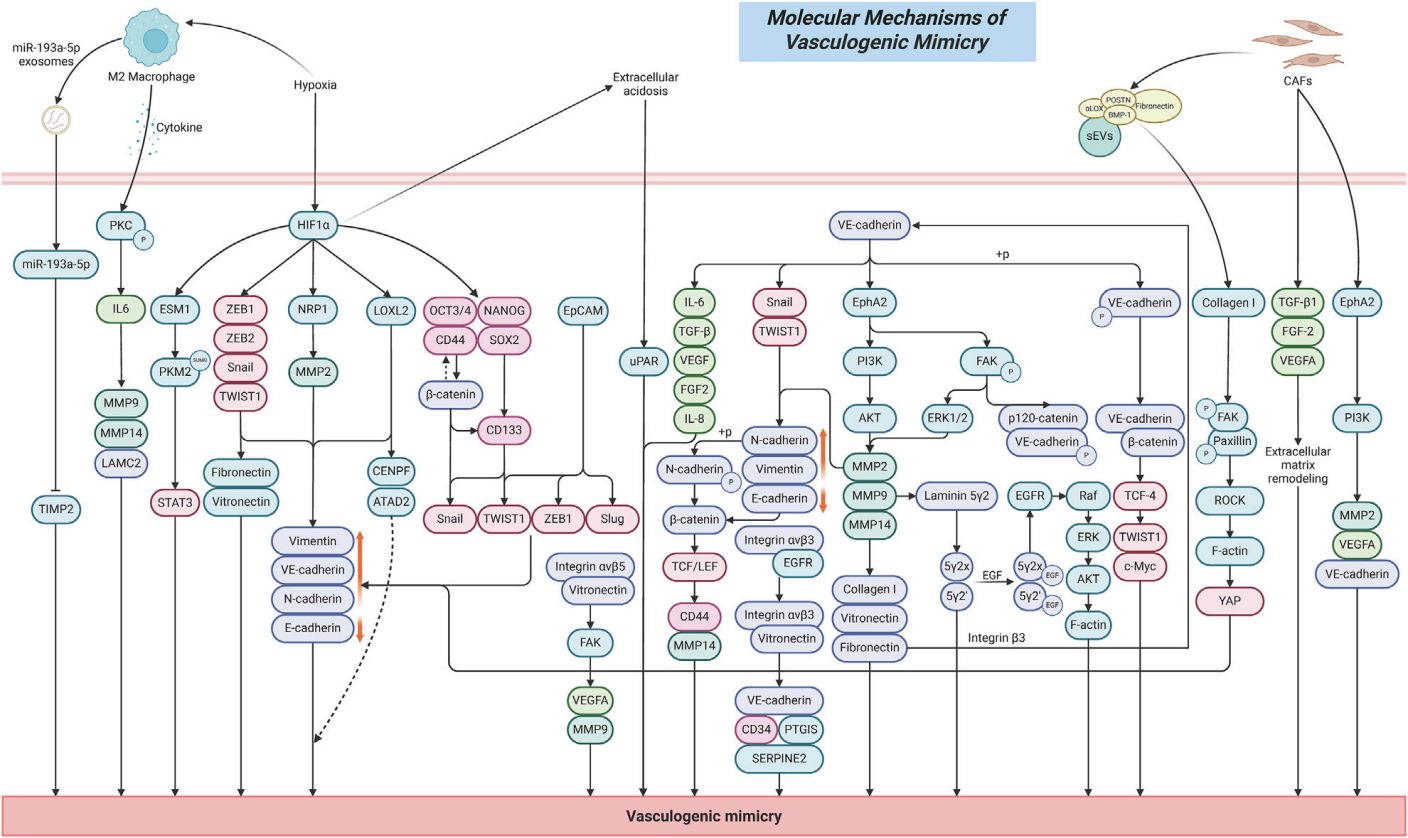
Schematic diagram of the molecular mechanisms underlying VM formation.

In addition, other CAMs such as N-cadherin, E-cadherin, integrin αvβ3, and integrin αvβ5 also contribute to the regulation of VM through multiple mechanisms, including mediating ECM degradation, remodeling cell morphology, maintaining the structural stability of VM channels, promoting intercellular signal transduction, and facilitating cell migration, invasion, and EMT (Wang et al., 2015; Cheng et al., 2019; Camorani et al., 2017; Ruffini et al., 2015). N-Cadherin is a calcium-dependent transmembrane glycoprotein and a key member of the CAMs family, primarily responsible for maintaining tissue integrity and polarity through mediating homophilic cell–cell adhesion (Blaschuk, 2015). Upregulation of N-Cadherin expression has been shown to significantly impair tumor cells’ ability to form tubular VM structures, along with concurrent reductions in their invasive, migratory, and proliferative capacities (Wang et al., 2015). Studies have also revealed that N-Cadherin facilitates transendothelial migration by promoting the nuclear translocation of β-catenin, which subsequently activates TCF/LEF-mediated transcription of CD44-a process that enhances the formation of VM (Qi et al., 2005; Resendiz-Hernández et al., 2024). E-Cadherin is a calcium-dependent cell adhesion molecule primarily expressed in epithelial cells, where it plays a crucial role in maintaining intercellular adhesion and tissue integrity (Shash et al., 2021). Reduced expression of E-Cadherin weakens cell–cell adhesion, making it easier for tumor cells to detach from the primary lesion, thereby creating a favorable environment for the formation of VM (Cheng et al., 2019). Further studies have shown that the cytoplasmic tail of E-Cadherin forms a complex with β-catenin and p120-catenin, participating in intracellular signaling pathways. Downregulation of E-Cadherin facilitates the nuclear translocation of β-catenin, thereby activating the Wnt/β-catenin signaling pathway, promoting EMT, and enhancing the plasticity and migratory capacity of tumor cells, which ultimately contributes to VM formation (Yu et al., 2019; Qi et al., 2015). Integrin αvβ3 and integrin αvβ5 are key cell adhesion receptors belonging to the integrin family and are extensively involved in the interaction between tumor cells and the ECM (Schittenhelm et al., 2013). Studies have shown that integrin αvβ3 forms a complex with EGFR on the cell membrane, which induces a conformational change in αvβ3, enhancing its binding affinity to ECM ligands such as vitronectin. This interaction further promotes the expression of VE-cadherin, CD34, and SERPINE2, thereby facilitating the formation of vessel-like network structures by tumor cells (Camorani et al., 2017). Disruption of this complex can inhibit tumor growth and cell proliferation, as well as reduce the formation of PAS^+^/CD31^-^ VM channels *in vivo*. Meanwhile, integrin αvβ5 promotes VM formation by binding to vitronectin in the ECM and activating the FAK signaling pathways, which subsequently upregulate VEGFA and MMP9 expression, enhancing tumor cell migration, invasion, and the formation of tubular VM structures (Ruffini et al., 2015). The molecular mechanisms underlying VM formation are illustrated in Figure 1.

### 2.2 ECM remodeling and activation of the proteolytic system

ECM remodeling refers to the dynamic changes in the composition, structure, and physicochemical properties of the ECM (Bonnans et al., 2014). It is a crucial mechanism involved in normal development, tissue repair, and the maintenance of homeostasis, but in disease conditions, ECM often undergoes abnormal or excessive remodeling, which can act as a driving force in pathological processes (Prakash and Shaked, 2024).

ECM remodeling plays a central driving role in tumor VM. It creates a critical microenvironment that facilitates the occurrence of VM by altering the biochemical composition, physical properties, and spatial architecture of tumor cells (Yuan et al., 2023). Firstly, differences in the expression of ECM components would directly mediate the formation of VM. Collagen I, as one of the key components of the ECM, can induce efficient formation of vascular-like network structures by cancer stem cells (CSCs) under low-content conditions in pancreatic ductal adenocarcinoma (Cannone et al., 2022). Conversely, as the proportion of Collagen I increases, the ability to form VM significantly decreases or even completely disappears. In addition, cancer-associated fibroblasts (CAFs) can enhance VM formation in coordination with the ECM by secreting factors such as TGF-β1, FGF-2, and VEGFA. The ECM components collagen I and fibronectin can enhance the proliferation, migration, and stemness of melanoma tumor cells by activating the Integrin β3/VE-cadherin/EphA2/PI3K signaling axis, upregulating MMP-2 expression, and inducing the EMT marker vimentin (Shuai et al., 2024). In xenograft tumors, they also significantly increase the number of CD31^-^/PAS^+^ channels, accompanied by MMP2–mediated matrix remodeling and microangiogenesis, thereby promoting the formation of VM tubular networks.

Secondly, during tumor development, highly invasive tumor cells can secrete large amounts of matrix metalloproteinases (MMPs), such as MMP-2, MMP-9, and MMP-14, while the overexpression of MMPs can promote the degradation of ECM components, thereby increasing tumor cell migration, invasion, and the capacity for VM formation (Seftor et al., 2001; Cabral-Pacheco et al., 2020; Najafi et al., 2019; Cai et al., 2019; Sun et al., 2007). Specifically, the activation of MMPs can promote ECM degradation by cleaving key ECM molecules such as collagen I, fibronectin, and vitronectin (Kenny and Lengyel, 2009). For example, MMP-2 can cleave laminin-5 into 80 kDa γ2x and 105 kDa γ2 fragments, which are rich in EGF-like domains. These fragments then bind to the epidermal growth factor receptor (EGFR), activating its downstream Raf/ERK/AKT signaling pathway, inducing F-actin cytoskeleton reorganization, and enhancing tumor cell migration and VM formation ability (Li et al., 2017). Meanwhile, MMPs can also contribute to tumor VM formation by acting downstream of other regulatory signaling pathways. EphA2, a receptor tyrosine kinase located on the cell surface, facilitates VM by activating the PI3K/Akt signaling pathway, which in turn upregulates MMP9 expression, thereby promoting ECM remodeling in tumors (Pandey et al., 1994; Tang et al., 2016). EphA2 can also activate the FAK signaling pathway, which subsequently reduces the phosphorylation levels of ERK1/2. This leads to decreased activity of MMP-2 and MT1-MMP, thereby affecting ECM remodeling and promoting the formation of VM (Lu et al., 2013; Hess and Hendrix, 2006).

In addition, CAFs directly remodel the ECM by secreting small extracellular vesicles (sEVs) enriched with active lysyl oxidase (αLOX) (Liu et al., 2023). This is primarily because sEVs can target and bind to collagen I via integrin α2β1, while αLOX is anchored to the vesicle surface by forming complexes with fibronectin (FN), periostin (POSTN), and bone morphogenetic protein 1 (BMP-1) located on the sEV membrane. αLOX then directly catalyzes collagen cross-linking and increases the levels of the mature cross-linking product pyridinoline (PYD), resulting in ECM stiffening (Liu et al., 2023; You et al., 2019; Jue et al., 2017a). This stiffened ECM microenvironment subsequently activates FAK/paxillin phosphorylation, induces ROCK-dependent actomyosin contraction, promotes YAP nuclear translocation, and drives the EMT process in tumor cells, thus influencing the occurrence of VM (Liu et al., 2023; Roy et al., 2024).

### 2.3 The relationship between CSCs characteristics and VM capability

CSCs are a special subpopulation of cells present in tumors. They are considered the primary driving force behind tumor initiation, progression, and metastasis, possessing a high capacity for self-renewal and multipotent differentiation (Zhou et al., 2023). CSCs possess the ability to evade immune surveillance and are not only the source of tumor heterogeneity but also closely associated with drug resistance and tumor recurrence (Duan et al., 2021). Studies have shown that tumor cells with VM capability often exhibit stem cell-like properties (Wechman et al., 2020). Through self-deformation and rearrangement, they form microvascular channel networks composed directly of tumor cells without the involvement of endothelial cells. These channels connect with the host vasculature to transport blood, thereby supplying oxygen and nutrients to the tumor interior (Liu et al., 2025). This enables tumor cells to survive under hypoxic and nutrient-deficient conditions, promoting continuous tumor growth and progression. This CSC-driven VM process is significantly associated with high tumor invasiveness, metastatic potential, and poor prognosis (Treps et al., 2021).

The maintenance of CSC stemness is closely associated with VM formation. SOX2, one of the core stemness transcription factors in tumors, is highly expressed in CSCs and plays a critical role in maintaining CSCs’ self-renewal, pluripotency, and drug resistance (Novak et al., 2020). Low expression of SOX2 significantly reduces the expression of the colorectal cancer stemness marker CD133 and inhibit the abilities of cancer cells to form spheroids and VM structures (Chen et al., 2020). This effect is mainly achieved by decreasing the expression of VE-cadherin, N-cadherin, Vimentin, Snail, and TWIST1, while increasing E-cadherin expression, which in turn suppresses tumor cell proliferation, migration, invasion, and the EMT process. OCT4 is one of the important markers of tumor stemness. Studies have shown that its positive expression level in tumor specimens from gallbladder cancer patients is significantly higher than in adjacent non-cancerous tissues, and it is significantly correlated with the occurrence of VM, metastasis, invasion, and TNM stage in gallbladder cancer patients (Zhang et al., 2018). CD44 is a transmembrane glycoprotein that is overexpressed in CSCs (Hassn Mesrati et al., 2021). In tissue samples from renal cancer patients, CD44 expression is significantly positively correlated with tumor microvessel density, size, grade, and stage, and it also shows a significant correlation with VM (Zhang et al., 2013). In breast cancer tissues, high expression of CD44^+^/CD24^-^ can increase the invasiveness of breast cancer cells and promote tumor recurrence and distant metastasis (Idowu et al., 2012). Further studies have shown that inhibiting the expression of CD44^+^/CD24^-^ can significantly suppress mammosphere formation and reduce the number of VM channels in breast cancer cells (Resendiz-Hernández et al., 2024). This effect is likely closely related to the inhibition of the Wnt/β-catenin signaling pathway, as well as a significant reduction in cell migration, invasion, and EMT capabilities (Wei et al., 2019). In addition, high expression of CD44 is also significantly positively correlated with the occurrence of VM in oral squamous cell carcinoma and mucoepidermoid carcinoma (Irani and Dehghan, 2018; Irani and Jafari, 2018).

The plasticity of CSCs is a crucial mechanism for their transformation into endothelial-like phenotypes. CSCs possess high plasticity that enables them to acquire endothelial-like characteristics through EMT and directly participate in the construction of VM structures (Wei et al., 2021). Studies have shown that epithelial cell adhesion molecule (EpCAM), as one of the key surface markers of breast cancer CSCs, induces the loss of E-cadherin and upregulates the expression of Vimentin and VE-cadherin by activating EMT-related transcription factors Slug, Twist1, and ZEB1 (Wu et al., 2019). This enhances cellular plasticity, enabling tumor cells to mimic endothelial phenotypes and form endothelial cell–independent VM channels.

CSCs are also capable of driving VM formation under stress conditions such as acidification and hypoxia in the tumor microenvironment by up-regulating the expression of the EMT transcription factors ZEB1/2, Snail, and Twist, and inhibiting E-cadherin, while promoting the expression of VE-cadherin, Fibronectin, and Vitronectin (Murai and Matsuda, 2023). This process depends on the activation of the PI3K-Akt pathway to enhance the activity of MMP2/14, which mediates the degradation of Laminin 5γ2 (Ln5γ2) to construct the VM network, and is synergistically reinforced by HIF-1α and microenvironmental signals such as VEGF/VEGFR2 and Ephrin-A2. Overall, during the EMT process, CSCs gradually lose the typical characteristics of epithelial cells, such as reduced intercellular tight junctions and decreased expression of the epithelial marker E-cadherin; at the same time, they acquire mesenchymal traits, including changes in cell morphology from an epithelial-like polygonal shape to a mesenchymal-like spindle shape-and increased expression of mesenchymal markers such as N-cadherin and Vimentin (Sun et al., 2017). This phenotypic transformation endows CSCs with enhanced migratory and invasive capabilities and lays the foundation for the formation of tumor VM.

### 2.4 Regulation of VM by the tumor microenvironment

Hypoxia is a common phenomenon in the tumor microenvironment, particularly in the inner growth regions of tumor (Ding et al., 2025). Studies have shown that under hypoxic conditions, tumor cells adapt to the low-oxygen environment by activating HIF-1α (Li et al., 2020). The activation of HIF-1α can induce tumor cells to exhibit endothelial-like characteristics (Wei et al., 2021). This transdifferentiation represents a significant phenomenon in tumor biology, known as tumor VM, which is the subject of our current investigation. For example, in the hypoxic microenvironment of ovarian cancer (OC), HIF-1α transcriptionally activates ESM1, which drives the SUMOylation of PKM2, promoting PKM2 dimerization and nuclear translocation (Zhang et al., 2024a). This process subsequently activates the STAT3 signaling pathway and metabolic reprogramming, including the Warburg effect and fatty acid synthesis, ultimately inducing the formation of VM.

In the hypoxic tumor microenvironment of HCC, HIF-1α is activated as a transcription factor and directly upregulates the expression of LOXL2 (Wang et al., 2017). This leads to the repression of E-cadherin and the promotion of Vimentin expression, thereby inducing EMT and enhancing tumor cell migration and invasion. Meanwhile, elevated LOXL2 expression can promote VE-cadherin expression, facilitating the formation of VM channels in HCC cells, providing a non-endothelial-dependent blood supply for the tumor. Moreover, the HIF-1α/LOXL2 axis further promotes tumor progression and metastasis by regulating oncogenes such as CENPF and ATAD2, activating mitotic and cell growth pathways, thereby synergistically amplifying the pro-VM effect of HIF-1α (Wang et al., 2017). In the hypoxic microenvironment of lung cancer (LUAD), HIF-1α transcriptionally upregulates NRP1 expression by directly binding to the NRP1 promoter region (−2009 to −2017 bp). This upregulation subsequently activates downstream signaling molecules, including VE-cadherin, MMP2, and Vimentin, promoting EMT and the formation of VM (Fu et al., 2021). Further clinical sample analysis confirmed a significant positive correlation between the HIF-1α/NRP1 axis and VM, with high expression levels of both HIF-1α and NRP1 predicting poor patient prognosis. Functional studies demonstrated that NRP1 is a key effector mediating HIF-1α-induced tumor migration, invasion, and VM formation, and that targeting the HIF-1α-induced overexpression of NRP1 can inhibit the malignant progression of LUAD (Fu et al., 2021). Moreover, hypoxia can enhance the stemness of tumor cells by upregulating CSC markers such as CD133, CD44, SOX2, OCT3/4, and NANOG, thereby activating the EMT process and promoting the formation of VM (Pietras et al., 2014; Iida et al., 2012; Bhuria et al., 2019).

Tumor cells preferentially generate energy through anaerobic glycolysis, resulting in the production of large amounts of lactate that lower the pH of the surrounding microenvironment and establish an acidic tumor microenvironment (Zhao et al., 2024). This acidic condition facilitates tumor cell invasion and metastasis, while also contributing to immune suppression (Böhme and Bosserhoff, 2016; Ganapathy-Kanniappan, 2017). Studies have shown that acidosis in the tumor microenvironment significantly enhances the expression of uPAR, which drives the formation of VM networks in melanoma cells and is associated with drug-resistant phenotypes (Andreucci et al., 2022). Acidosis can also reversibly inhibit the formation of capillary networks and the invasive ability of endothelial cells, while promoting VM in tumor cells as an alternative vascularization pathway (Andreucci et al., 2022; Andreucci et al., 2021). Further studies have found that acidosis can induce a metabolic shift in tumor cells from glycolysis to oxidative phosphorylation, relying on fatty acid oxidation and glutamine metabolism, which may be one of the key reasons supporting the energy demand for VM formation (Peppicelli et al., 2016; Corbet et al., 2016; Andreucci et al., 2022).

Inflammatory cells and cytokines in the tumor microenvironment play crucial roles in regulating VM through CSCs. Studies have shown a significant positive correlation between CD163^+^, a marker of M2 tumor-associated macrophages (TAMs), and VM channel density in glioma tissue specimens (Zhang et al., 2017). This is primarily because M2 TAMs induce a marked upregulation of IL-6 expression in glioma cells via activation of protein kinase C (PKC) phosphorylation, thereby promoting the formation of VM tubular structures and upregulating key markers such as MMP9, MMP14, and LAMC2. In the microenvironment of renal cell carcinoma (RCC), TAMs transcriptionally upregulate miR-193a-5p via HIF-1α (Liu et al., 2022). The upregulated miR-193a-5p is subsequently packaged into exosomes and targets the 3′ untranslated region (3′UTR) of tissue inhibitor of metalloproteinases 2 (TIMP2), leading to decreased TIMP2 expression. This downregulation alleviates the inhibitory effect of TIMP2 on VM-related molecules, thereby significantly enhancing the VM-forming capacity and invasiveness of tumor cells. In addition, it has been found that CAFs can promote the formation of VM by secreting active factors that activate the membrane receptor EphA2 on gastric cancer cells, consequently triggering a PI3K/AKT signaling-dependent mechanism (Kim et al., 2019). The synergistic actions of these molecular mechanisms position TAMs and CAFs as pivotal drivers of VM formation. By orchestrating the secretion of diverse factors and activating multiple signaling pathways, they effectively regulate VM formation and function, providing a critical alternative blood supply route for highly invasive tumors, while promoting malignant phenotypic progression and therapy resistance.

## 3 Mechanisms of NPs and TCM in regulating tumor VM

### 3.1 Regulatory mechanisms of lung cancer

**FIGURE 2:**
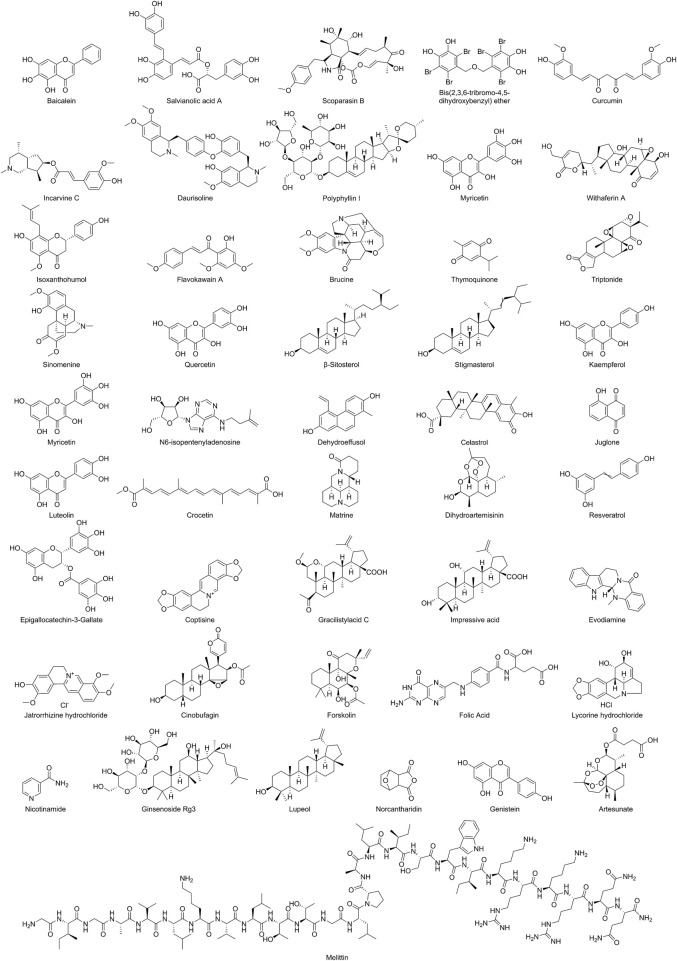
Schematic representation of the chemical structures of NPs.

**FIGURE 3:**
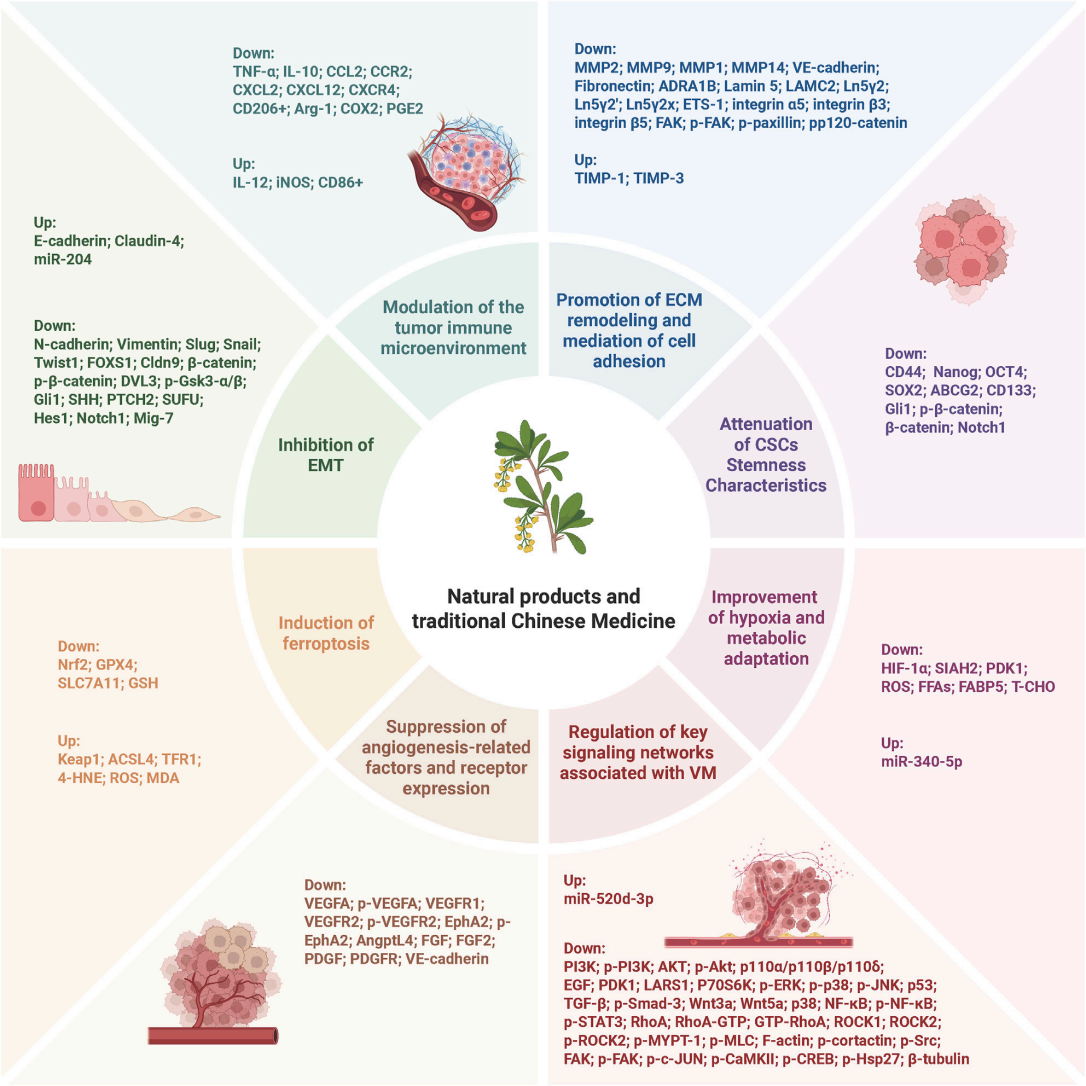
Schematic representation of the molecular mechanisms by which NPs and TCM inhibit tumor VM. The mechanisms include inhibition of EMT, modulation of tumor immune microenvironment, promotion of ECM remodeling and cell adhesion, attenuation of CSC stemness characteristics, induction of ferroptosis, suppression of angiogenesis-related factors and receptor expression, improvement of hypoxia and metabolic adaptation, and regulation of key signaling networks associated with VM.

Baicalein, as an active component of TCM, is mainly derived from the root extract of Scutellaria baicalensis, a plant belonging to the Lamiaceae family (the chemical structures of NPs are shown in Figure 2). Zhe et al. reported that in the Non-Small Cell Lung Cancer (NSCLC) A549 cell model, baicalein inhibits the RhoA/ROCK signaling pathway in a dose-dependent manner, disrupts the cytoskeletal F-actin network, downregulates the expression of VM-related factors including VE-cadherin, EphA2, MMP2/9/14, PI3K, and LAMC2, and blocks the interaction between tumor cells and the ECM, thereby suppressing the formation of VM tubular structures (Zhang et al., 2020). *In vivo* studies demonstrated that baicalein treatment markedly decreased the number of PAS-positive VM channels in Balb/c nude mouse xenograft models, accompanied by significant tumor growth inhibition and reduced phosphorylation of myosin light chain (MLC). The effects were comparable to those observed with the ROCK inhibitor fasudil, providing strong evidence that the RhoA/ROCK signaling pathway serves as a critical target mediating the anti-VM effects of baicalein (detailed molecular mechanisms are shown in Table 1 and Figure 3).

**TABLE 1:** Molecular mechanisms of NPs and TCM inhibiting tumor VM.

Tumor types	Main source	Active components	Signaling pathway	Molecular mechanisms	Experimental model	Concentration	References
Lung cancer	Scutellaria baicalensis	Baicalein	RhoA/ROCK	*In vitro*: RhoA↓, ROCK1/2↓, VE-cadherin↓, EphA2↓, MMP2/9/14↓, LAMC2↓, PI3K↓, F-actin↓ *In vivo*: ROCK1/2↓, p-MLC↓, CD34-/PAS+↓	*In vitro*: A549 cells *In vivo*: BALB/c-nude mice	*In vitro*: 15–60 μM *In vivo*: 10 mg/kg	Zhang et al. (2020)
	Salvia miltiorrhiza	Salvianolic acid A	PI3K/Akt/mTOR	*In vitro*: p-PI3K↓, p-Akt↓, p-mTOR↓, MMP2↓, VE-cadherin↓, EphA2↓	*In vitro*: NCI-H1299/A549 cells	*In vitro*: 5–50 μM	Jin et al. (2021)
	Marine-derived fungus, Eutypella sp. F0219	Scoparasin B	VEGFA/VEGFR2	*In vitro*: VE-cadherin↓, N-cadherin↓, Vimentin↓, Snail↓, E-cadherin↑, MMP9↓, VEGFA↓, p-VEGFR2↓ *In vivo*: PAS^+^↓, CD31↓, VEGF-A↓	*In vitro*: NCI-H1299 cells *In vivo*: Nude mice	*In vitro*: 5–15 μM *In vivo*: 0.75–1.5 mg/kg	Lin et al. (2023)
	Symphyocladia latiuscula	Bis (2,3,6tribromo-4,5-dihydroxybenzyl) ether	N/A	N/A	*In vitro*: A549 cells	*In vitro*: 2.5–10 μM	Dong et al. (2021)
	YFJDT	N/A	N/A	*In vitro*: HIF1α↓, VE-cadherin↓, N-cadherin↓, Vimentin↓, E-cadherin↑, SLC7A11↓, GPX4↓, ACSL4↑, TFR1↑, 4-HNE↓ *In vivo*: CD34^-^/PAS^+^↓, VE-cadherin↓	*In vitro*: A549 cells *In vivo*: BALB/c-nude mice	*In vitro*: Drug-containing serum *In vivo*: 9–36 g/kg	Wang et al. (2025c)
Hepatocellular Carcinoma	Paris polyphylla	Polyphyllin I	PI3K/Akt/TWIST1/VE-cadherin	*In vitro*: VE-cadherin↓, VEGFR1/2↓, MMP2/9↓, Lamin 5↓, Vimentin↓, TWIST1↓, E-cadherin↑, p-Akt↓, CD34↓, CD133↓ *In vivo*: CD34-/PAS+↓, VE-cadherin↓, VEGFR1/2↓, MMP2/9↓, Lamin 5↓, Vimentin↓, TWIST1↓, E-cadherin↑, p-Akt↓	*In vitro*: SMMC7721/PLC cells *In vivo*: BALB/c-nude mice/Liver cancer patients	*In vitro*: 0.1–1 μM *In vivo*: 10 mg/kg (BALB/c-nude mice)/1 mg/kg (Liver cancer patients)	Xiao et al. (2018)
	Curcuma longa	Curcumin	PI3K/Akt、STAT3	*In vitro*: p-Akt↓, p-STAT3↓, MMP9 ↓	*In vitro*: SK-Hep-1 cells	*In vitro*: 3–30 μM	Chiablaem et al. (2014)
	Incarvillea Sinensis	Incarvine C	ROCK/MYPT-1	*In vitro*: p-MYPT-1↓, VE-cadherin↓, EphA2↓, PI3K↓, MMP2/9/14↓, LAMC2↓	*In vitro*: MHCC97H cells	*In vitro*: 7.5–50 μM	Zhang et al. (2015)
	Bee venom	Melittin	HIF-1 α/Akt	*In vitro*: HIF1α↓, VEGF↓, MMP2/9↓, N-cadherin↓, Vimentin↓, E-cadherin↑, p-Akt↓ *In vivo*: CD34-/PAS+↓, HIF-1 α↓	*In vitro*: SMMC-7721 cells *In vivo*: BALB/c-nude mice	*In vitro*: 2–4 μg/mL *In vivo*: 50–100 μg/kg	Chen et al. (2019)
	Celastrus orbiculatus	Celastrus orbiculatus extract	Notch1/Hes1	*In vitro*: Notch1↓, Hes1↓, Vimentin↓, E-cadherin↑ *In vivo*: CD34-/PAS+↓, Notch1↓, Hes1↓	*In vitro*: MHCC97-H/HepG2 cells *In vivo*: BALB/c-nude mice	*In vitro*: 20–80 μg/mL *In vivo*: 20–80 mg/kg	Jue et al. (2017b)
	Celastrus orbiculatus	Celastrus orbiculatus extract	EphA2	*In vitro*: EphA2↓, MMP2/9↓, E-cadherin↑, VE-cadherin↓, TWIST1↓ *In vivo*: CD31-/PAS+↓, EphA2↓, MMP2/9↓, E-cadherin↑, VE-cadherin↓, TWIST1↓	*In vitro*: HepG2/MHCC97-H cells *In vivo*: BALB/c-nude mice	*In vitro*: 20–80 μg/mL *In vivo*: 20–80 mg/kg	Chu et al. (2021)
	Celastrus orbiculatus	Celastrus orbiculatus extract	EphA2/PI3K/FAK/VE-CAD	*In vitro*: EphA2↓, E-cadherin↑, MMP2/9↓, N-cadherin↓, TWIST1↓, p-PI3K↓, p-FAK↓, VE-cadherin↓	*In vitro*: HepG2/MHCC97-H cells	*In vitro*: 80 μg/mL	Chen et al. (2024)
	Rhizoma Menispermi	Daurisoline	RhoA/ROCK2/Akt and ERK-p38 MAPK	*In vitro*: p-MYPT-1↓, p-MLC-2↓, ROCK2↓, VE-cadherin↓, VEGFR2↓, p-Akt↓, p-ERK1/2↓, p-p38↓ *In vivo*: CD34-/PAS+↓, ROCK2↓, RhoA-GTP↓,VE-cadherin↓, VEGFR2↓, p-Akt↓, p-ERK1/2↓, p-p38↓	*In vitro*: QGY-7703/MHCC97H cells *In vivo*: BALB/c-nude mice	*In vitro*: 5–20 μM *In vivo*: 20 mg/kg	Zhang et al. (2021c)
	Various fruits and vegetables	Myricetin	PAR1	*In vitro*: PAR1↓, VE-cadherin↓, E-cadherin↑, VEGFR1/2↓, HIF-1α↓, TWIST1↓ *In vivo*: CD31-/PAS+↓, E-cadherin↑, VEGFR1/2↓	*In vitro*: HepG2/SMMC-7721 cells *In vivo*: BALB/c-nude mice	*In vitro*: 20–80 μM *In vivo*: 15–30 mg/kg	Wang et al. (2022a)
	Withania somnifera	Withaferin A	Keap1/Nrf2	*In vitro*: Keap1↑, Nrf2↓, N-cadherin↓, E-cadherin↑, VE-cadherin↓, SLC7A11↓, ROS↑, MDA↑, GSH↓	*In vitro*: HepG2/SNU449 cells	*In vitro*: 10 μM	Zhang et al. (2023)
	Chloranthus henryi	Flavokawain A	CXCL12/PI3K/Akt/HIF-1α/NF-κB/TWIST1	*In vitro*: CXCL12↓, CXCR4↓, p-PI3K↓, p-Akt↓, NF-κB↓, HIF-1α↓, TWIST1↓, VE-cadherin↓, Vimentin↓, Snail1↓, E-cadherin↑ *In vivo*: CD31-/PAS+↓, E cadherin↑, VE cadherin↓, Vimentin↓, p-Akt↓, TWIST1↓	*In vitro*: SMMC-7721/HepG2 cells *In vivo*: BALB/c-nude mice	*In vitro*: 10–40 μM *In vivo*: 30–120 mg/kg	Xiao et al. (2023)
	Trichoderma harzianum	Isoxanthohumol	TGF-β/Smad	*In vitro*: MMP9↓, PDGF↓, AngptL4↓	*In vitro*: MDA-MB-231、HepG2 cells	*In vitro*: 54.6–141 μM	Serwe et al. (2012)
	Biejiajian Pills	N/A	RhoA/ROCK	*In vitro*: RhoA↓, ROCK1↓, VE-cadherin↓, PI3K↓	*In vitro*: HepG2 cells	*In vitro*: Drug-containing serum	An et al. (2018)
	Compound Taxus capsule	N/A	N/A	*In vitro*: MMP9↓, E-cadherin↑, N-cadherin↓, Vimentin↓ *In vivo*: VEGFA↓	*In vitro*: SMMC-7721/Bel7402 cells *In vivo*: BALB/c-nude mice	*In vitro*: Drug-containing serum *In vivo*: 300 mg/kg	Gao et al. (2024)
Breast Cancer	Grape seeds	Grape seed proanthocyanidins	N/A	*In vitro*: TWIST1↓, VE-cadherin↓, E-cadherin↑	*In vitro*: HCC1937 cells	*In vitro*: 100–200 μg/mL	Luan et al. (2015)
	Arthrospira platensis	Phycocyanin	MAPK/ERK	*In vitro*: p-ERK1/2↓, COX2↓, PGE2↓, VEGFR2↓, MMP9↓	*In vitro*: MDA-MB-231 cells	*In vitro*: 3 μM	Ravi et al. (2015)
	Loganiaceae	Brucine	EphA2/MMPs	*In vitro*: EphA2↓, MMP2/9↓, F-actin↓, β-tubulin↓	*In vitro*: MDA-MB-231 cells	*In* *vitro*: 0.0625–1 mM	Xu et al. (2019)
	Nigella sativa Linn	Thymoquinone	PI3K/Akt, Wnt3a	*In vitro*: p-Akt/Akt↓, Wnt3a↓, VE-cadherin↓, MMP2/9↓, VEGF↓, EGF↓, FGF↓	*In vitro*: MDA-MB-231 cells	*In vitro*: 10–30 μM	Haiaty et al. (2021)
	Tripterygium wilfordii	Triptonide	N/A	*In vitro*: TWIST1↓, Notch1↓, p-NF-κB↓,VE-cadherin↓, VEGFR2↓, N-cadherin↓ *In vivo*: TWIST1↓	*In vitro*: MDA-MB-231/MDA-MB-468 cells *In vivo*: NOD-SCID mice	*In vitro*: 2.5–10 nM *In vivo*: 3 mg/kg	Zhang et al. (2021a)
	Sinomenium acutum	Sinomenine	miR-340-5p/SIAH2/HIF-1α	*In vitro*: miR-340-5p↑, SIAH2↓, HIF-1α↓, EphA2↓, VE-cadherin↓, MMP9↓, E-cadherin↑, Snail↓, N-cadherin↓	*In vitro*: MDA-MB-231 cells	*In vitro*: 0.25–1 mM	Song et al. (2022)
	Curcuma longa	Curcumin	PI3K/Akt, MAPK	*In vitro*: p-Akt↓, p-CREB↓, p-Gsk3-α/β↓, p-ERK1/2↓, p53↓, p38↓, p-JNK↓, p-Hsp27↓, P70S6K↓	*In vitro*: MBCDF-T/HCC1806 cells	*In vitro*: 20 μM	Morales-Guadarrama et al. (2022)
	Green tea	Epigallocatechin gallate	STAT3	*In vitro*: p-STAT3↓, Fibronectin↓	*In vitro*: MDA-MB-231 cells	*In vitro*: 30 μM	Gonzalez Suarez et al. (2021)
	Xian-ling-lian-xia-fang	Quercetin, Kaempferol, Stigmasterol, β - Sitosterol, etc	VEGF/MMPs	*In vitro*: VEGFA↓, MMP2/9↓, Vimentin↓, VE-cadherin↓, TWIST1↓, E-cadherin↑, TIMP-1↑, TIMP-3↑, F-actin↓ *In vivo*: CD34-/PAS+↓, VEGFA↓, MMP2/9↓, Vimentin↓, VE-cadherin↓, TWIST1↓, E-cadherin↑, TIMP-1↑, TIMP-3↑	*In vitro*: MDA-MB-231 cells *In vivo*: BALB/c-nude mice	*In vitro*: 50–200 μg/mL *In vivo*: 18 g/kg	Li et al. (2022a)
Glioblastoma	Various fruits and vegetables	Myricetin	PI3K/Akt, JNK	*In* *vitro*: p-Akt↓, p110α/p110β/p110δ↓, PDK1↓, p-c-JUN↓, p-ROCK2↓, p-paxillin↓, p-cortatin↓, VE-cadherin↓	*In* *vitro*: U-87 cells	*In* *vitro*: 5–20 μM	Zhao et al. (2018)
	Curcuma longa	Curcumin	EphA2/PI3K/MMP-2	*In* *vitro*: EphA2↓, PI3K↓, MMP2↓	*In* *vitro*: U251 cells	*In* *vitro*: 5–40 μM	Liang et al. (2014)
	Tripterygium wilfordii	Celastrol	PI3K/Akt/mTOR	*In* *vitro*: VE-cadherin↓, HIF-1α↓, p-PI3K↓, p-Akt↓, p-mTOR↓ *In* *vivo*: CD31-/PAS+↓, EphA2↓, VECadherin↓, HIF-1α↓, p-PI3K↓, p-Akt↓, p-mTOR↓	*In* *vitro*: U87/U251 cells *In* *vivo*: BALB/c-nude mice	*In* *vitro*: 0.25–1 mM *In* *vivo*: 0.5–2 mg/kg	Zhu et al. (2020)
	N/A	N6-isopentenyladenosine	Src/p120-catenin	*In* *vitro*: p-Src↓, pp120-catenin↓, VE-cadherin↓, E-cadherin↓, p-β-catenin↓, pGSKα/β↓, GTP-RhoA↓, F-actin↓	*In* *vitro*: U87/U343/U251 cells	*In* *vitro*: 10 μM	Pagano et al. (2021)
	Walnut trees	Juglone	VEGFA/VEGFR2/Akt/SNAIL	*In* *vitro*: VE-cadherin↓, p-VEGFA↓, p-VEGFR2↓, p-Akt↓, Vimentin↓, Snail↓, E-cadherin↑ *In* *vivo*: CD31-/PAS+↓, VEGFA↓, VE-cadherin↓	*In* *vitro*: U251 cells *In* *vivo*: BALB/c-nude mice	*In* *vitro*: 10–20 μM *In* *vivo*: 0.5–1 mg/kg	Luo et al. (2024)
	Various traditional Chinese medicines	Stigmasterol	N/A	*In* *vitro*: FABP5↓, ADRA1B↓, FFAs↓, T-CHO↓	*In* *vitro*: U87 cells	*In* *vitro*: 240 μM	Wei et al. (2024)
	Ruta graveolens	Ruta graveolens water extract	N/A	*In* *vitro*: VEGF↓	*In* *vitro*: U87/U251/C6/patient-derived GBM CSCs cells	*In* *vitro*: 1 pg/mL-1 mg/mL	Camerino et al. (2024)
Gastric Cancer	Juncus effusus	Dehydroeffusol	N/A	*In* *vitro*: VE-cadherin↓, β-catenin↓, MMP2↓, p-ERK↓ *In* *vivo*: VE-cadherin↓, MMP2↓	*In* *vitro*: SGC-7901/AGS cells *In* *vivo*: BALB/c-nude mice	*In* *vitro*: 12–48 μM *In* *vivo*: 40–120 μg	Liu et al. (2015)
	Various fruits and green plants	Luteolin	Notch1/VEGF	*In* *vitro*: Notch1↓, VEGF↓	*In* *vitro*: Hs-746T/MGC-803 cells	*In* *vitro*: 30 μM	Zang et al. (2017)
	Saffron stigma	Crocetin	SHH	*In* *vitro*: SHH↓, PTCH2↓, SUFU↓, Gli1↓, F-actin↓	*In* *vitro*: NCI-N87/Hs-746T cells	*In* *vitro*: 30–100 μM	Zang et al. (2021)
	Artemisia annularis	Dihydroartemisinin	FGF2/FGFR1	*In* *vitro*: FGF2↓, TWIST1↓, MMP2↓, Vimentin↓, VE-cadherin↓, MMP9↓, N-cadherin↓ *In* *vivo*: CD34-/PAS+↓, FGF2↓, TWIST1↓, MMP2↓, Vimentin↓, VE-cadherin↓, MMP9↓, N-cadherin↓	*In* *vitro*: SGC-7901/HGC-27 cells *In* *vivo*: BALB/c-nude mice	*In* *vitro*: 40 μM *In* *vivo*: 50–100 mg/kg	Wang et al. (2024a)
	Xiaotan Sanjie Recipe	N/A	N/A	*In* *vivo*: CD34-/PAS+↓, MMP-2↓, MMP-9↓	*In* *vivo*: BALB/c-nude mice	*In* *vivo*: 0.4 mL/pcs	Zhou et al. (2011)
Prostate Cancer	Green tea	Epigallocatechin-3-Gallate	TWIST/VE-Cadherin/Akt	*In* *vitro*: TWIST↓, VE-cadherin↓, N-cadherin↓, Vimentin↓, p-Akt↓, Akt↓	*In* *vitro*: PC-3 cells	*In* *vitro*: 10–40 μM	Yeo et al. (2020)
	Red wine/grapes/berries/peanuts	Resveratrol	EphA2/TWIST/VE-cadherin/Akt	*In* *vitro*: p-EphA2↓, TWIST↓, VE-cadherin↓, p-Akt↓, MMP2↓, LAMC2↓	*In* *vitro*: PC-3/DU145 cells	*In* *vitro*: 10–20 μM	Han et al. (2022)
	Various fruits and vegetables	Kaempferol	N/A	N/A	*In* *vitro*: PC-3 cells	*In* *vitro*: 5–15 μM	Da et al. (2019)
	A variety of plants and daily diets	Luteolin and Quercetin	JNK/c-Jun	*In* *vitro*: MMP9↓, CD44↓, ABCG2↓, SOX2↓, Nanog↓, p-JNK↓, p-c-JUN↓	*In* *vitro*: Du145-III cells	*In* *vitro*: 20 μM	Tsai et al. (2016)
	Qilan Capsules	N/A	HIF-1α/VEGFα/MMP1	*In* *vitro*: HIF-1α↓,VEGF-α↓,MMP1↓	*In* *vitro*: PC-3 cells	*In* *vitro*: 0.7 g/mL	Yu et al. (2018)
Pancreatic Cancer	Ginseng	Ginsenoside Rg3	VE-cadherin/EphA2/MMP9/MMP2	*In* *vitro*/*vivo*: VE-cadherin↓, EphA2↓, MMP9↓, MMP2↓ *In* *vivo*: CD31-/PAS+↓, VE-cadherin↓, EphA2↓, MMP9↓, MMP2↓	*In* *vitro*: SW-1990 cells *In* *vivo*: BALB/c-nude mice	*In* *vitro*: 25–200 μM *In* *vivo*: 5–20 mg/kg	Guo et al. (2014)
	Ginseng	Ginsenoside Rg3	miR-204/DVL3/Wnt/β-catenin/PI3K/Akt	*In* *vitro*: miR-204↑, DVL3↓, E-cadherin↑, N-cadherin↓, vimentin↓, SOX2↓, OCT4↓, Nanog↓, β-catenin↓, p-Akt↓, p-PI3K↓ *In* *vivo*: CD31-/PAS+↓	*In* *vitro*: SW-1990/PCI-35 cells *In* *vivo*: BALB/c-nude mice	*In* *vitro*: 50 μM (SW-1990 cells) 10 μM (PCI-35 cells) *In* *vivo*: 10–20 mg/kg	Cai et al. (2024)
	Ginseng	Ginsenoside Rg3	HIF-1α/LARS1/mTOR	*In* *vitro*: VE-cadherin↓, VEFG↓, p-mTOR↓, HIF-1α↓, LARS1↓ *In* *vivo*: CD31-/PAS+↓, p-mTOR↓, HIF-1α↓, LARS1↓	*In* *vitro*: PANC-1/BxPC-3 cells cells *In* *vivo*: BALB/c-nude mice	*In* *vitro*: 50 μM *In* *vivo*: After pretreatment with 50 μM Rg3 for 24 h	Zhao et al. (2025)
	Tripterygium wilfordii	Triptonide	VE-cadherin/CXCL2	*In* *vitro*: VE-cadherin↓, CXCL2↓ *In* *vivo*: VE-cadherin↓	*In* *vitro*: Patu8988/Panc1 cells *In* *vivo*: BALB/c-nude mice	*In* *vitro*: 5–20 nM *In* *vivo*: 0.1 μg	Han et al. (2018)
Osteosarcoma	Coptidis rhizoma	Coptisine	N/A	*In* *vitro*: VE-cadherin↓, integrin β_3_↓, p-STAT3↓, VEGFA↓, MMP2↓, integrin α_5_↓, integrin β_5_↓	*In* *vitro*: MG63 cells	*In* *vitro*: 5–20 μM	Yu et al. (2014)
	Paris polyphylla	Paris polyphylla ethanol extract	N/A	*In* *vitro*: FAK↓, MIG-7↓, MMP2↓, MMP9↓ *In* *vivo*: CD31-/PAS+↓, FAK↓, MIG-7↓, MMP2↓, MMP9↓	*In* *vitro*: 143B cells *In* *vivo*: BALB/c-nude mice	*In* *vitro*: 2.5–7.5 μg/mL *In* *vivo*: 50–200 mg/kg	Yao et al. (2017)
	Acanthopanax genus	Gracilistylacid C/Impressic acid	N/A	N/A	*In* *vitro*: 143B cells	*In* *vitro*: 10 μM	Li et al. (2022b)
	Rhizoma Paridis	Rhizoma Paridis saponins	miR-520d-3p/MIG-7	*In* *vitro*: miR-520d-3p↑, MIG-7↓, p-PI3K↓, p-Akt↓, p-mTOR↓, MMP2/14↓, Ln5γ2’↓, Ln5γ2x↓, Ln5γ2↑, F-actin↓ *In* *vivo*: miR-520d-3p↑, MIG-7↓, p-PI3K↓, p-Akt↓, p-mTOR↓, MMP2/14↓	*In* *vitro*: 143B/MG63 cells *In* *vivo*: BALB/c-nude mice	*In* *vitro*: 0.25–1 μg/mL *In* *vivo*: 25–100 mg/kg	Yao et al. (2022)
Colorectal Cancer	Evodia rugulosa	Evodiamine	HIF-1α/VE-cadherin/VEGF/MMPs	*In* *vitro*: HIF-1α↓, VE-cadherin↓, VEGF↓, MMP2/9↓ *In* *vivo*: CD31-/PAS+↓, HIF-1α↓, VE-cadherin↓, VEGF↓, MMP2/9↓	*In* *vitro*: HCT-116 cells *In* *vivo*: BALB/c-nude mice	*In* *vitro*: 1.5 μM *In* *vivo*: 10 mg/kg	Zeng et al. (2021)
	Sophora alopecuroides Linn/Sophora tonkinensis	Matrine	Cldn9/MAPK	*In* *vitro*: Cldn9↓, p-JNK↓, p-ERK↓, E-cadherin↑, N-cadherin↓, MMP2/9↓, F-actin↓	*In* *vitro*: CT-26 cells	*In* *vitro*: 0.25–1 mM	Du et al. (2023)
	Astragalus Atractylodes mixture	N/A	ROS/HIF-1α/MMP2	*In* *vitro*: ROS↓, HIF-1a↓, MMP2↓	*In* *vitro*: HCT-116/LoVo cells	*In* *vitro*: 2.5–10 mg/mL	Zong et al. (2020)
	Yi Ai Fang	N/A	N/A	*In* *vitro*: HIF-1α↓, E-cadherin↑, Claudin-4↑, Vimentin↓ *In* *vivo*: CD31-/PAS+↓, HIF-1α↓, E-cadherin↑, Claudin-4↑, Vimentin↓	*In* *vitro*: HCT-116 cells *In* *vivo*: BALB/c-nude mice	*In* *vitro*: 25–100 μg/mL *In* *vivo*: 8–32 mg/mL	Hou et al. (2016)
Ovarian Cancer	Camellia sinensis	Gallated Catechins	TGF-β/Smad3	*In* *vitro*: p-Smad-3↓, p-p38↓, Snail↓, Slug↓, Fibronectin↓, Vimentin↓, MMP2↓	*In* *vitro*: ES-2 cells	*In* *vitro*: 30 μM	Sicard et al. (2021)
	*Bufo gargarizans*	Cinobufagin	FOXS1/CCL2/CCR2	*In* *vitro*: FOXS1↓, CD206^+^↓, TGF-β↓, CCL2↓, CCR2↓, LAMC2↓, MMP9/14↓ *In* *vivo*: CD31-/PAS+↓, LAMC2↓, MMP9/14↓, CCL2↓, CCR2↓, CD86^+^↑, iNOS↑, IL-12↑, CD206^+^↓,Arg-1↓, IL-10↓	*In* *vitro*: Skov3 cells *In* *vivo*: BALB/c-nude mice	*In* *vitro*: 5 μg/mL *In* *vivo*: 1.5–6 mg/kg	Wang et al. (2025a)
Choriocarcinoma	Coleus forskohlii	Forskolin	cAMP/Notch-1	*In* *vitro*: NICD↑, Hes1↑, E-cadherin↓, CK19↓, N-cadherin↑, Vimentin↑, ZEB1↑, VE-cadherin↑, EphA2↑, MMP2/9↑ *In* *vivo*: CD31-/PAS+↑, NICD↑, VE-cadherin↑, MMP2/9↑	*In* *vitro*: JAR/JEG-3 cells *In* *vivo*: BALB/c-nude mice	*In* *vitro*: 100 μM *In* *vivo*: 5 mg/kg	Xue et al. (2020)
Esophageal Squamous Cell Carcinoma	Green leafy vegetables/animal livers	Folic Acid	N/A	*In* *vitro*: EphA2↓	*In* *vitro*: ECA-109 cells	*In* *vitro*: 820 μg/mL	Xu et al. (2022)
	Aidi injection	N/A	N/A	*In* *vitro*: VEGFA↓, E-cadherin↑, N-cadherin↓, Vimentin↓ *In* *vivo*: VEGFA↓, E-cadherin↑, Vimentin↓	*In* *vitro*: EC9706/KYSE70 cells *In* *vivo*: BALB/c-nude mice	*In* *vitro*: 6–24 mg/mL *In* *vivo*: 1–4 g/kg	Shi et al. (2018)
	Modified Tongyou decoction	N/A	NF-κB/HIF-1α	*In* *vitro*: HIF-1α↓, NF-κB↓, VE-cadherin↓, TNF-α↓, Snail↓, MMP2/9↓, Vimentin↓, E-cadherin↑	*In* *vitro*: TE-1 cells	*In* *vitro*: 2,550 μg/mL	Wang et al. (2025b)
Cutaneous Melanoma	Lycoris radiata	Lycorine hydrochloride	N/A	*In* *vitro*: VE-cadherin↓ *In* *vivo*: VE-cadherin↓	*In* *vitro*: C8161 cells *In* *vivo*: BALB/c-nude mice	*In* *vitro*: 1.56–25 μM *In* *vivo*: 7.5–15 μg	Liu et al. (2012b)
	Animals/plants/microorganisms	Nicotinamide	N/A	*In* *vitro*: VE-cadherin↓, VEGFA↓	*In* *vitro*: C8161 cells	*In* *vitro*: 5–20 mM	Itzhaki et al. (2013)
	Coptis chinensis	Jatrorrhizine hydrochloride	N/A	*In* *vitro*: VE-cadherin↓, *In* *vivo*: N/A	*In* *vitro*: C8161 cells *In* *vivo*: BALB/c-nude mice	*In* *vitro*: 80–320 μM *In* *vivo*: 50 mg/kg	Liu et al. (2013)
	Spondias cytherea	Spondias cytherea Fruit Extract	NF-κB/COX-2	*In* *vitro*: CD133↓, NF-κB↓, COX-2↓, Vimentin↓, E-cadherin↑ *In* *vivo*: PAS↓, Vimentin↓, E-cadherin↑, CD133↓	*In* *vitro*: B16-F10 cells *In* *vivo*: C57BL/6J mice	*In* *vitro*: 2–10 mg/mL *In* *vivo*: 450–900 mg/kg	Yolande et al. (2018)
	Various fruits/vegetables/plants	Lupeol	N/A	*In* *vitro*: CD133↓, VE-cadherin↓ *In* *vivo*: CD31-/PAS+↓, CD133↓, VE-cadherin↓	*In* *vitro*: B16-F10 cells *In* *vivo*: C57BL/6J mice	*In* *vitro*: 100 μM *In* *vivo*: 40 mg/kg	Bhattacharyya et al. (2019)
	Piper capense	Piper capense fruit extract	N/A	*In* *vitro*: Vimentin↓, CD133↓, E-cadherin↑ *In* *vivo*: CD31-/PAS+↓, Vimentin↓, CD133↓, E-cadherin↑	*In* *vitro*: B16-F10 cells *In* *vivo*: C57BL/6J mice	*In* *vitro*: 100 μg/mL *In* *vivo*: 50–250 mg/kg	Wamba et al. (2021)
	Gallus gallus	Chicken embryo extract	N/A	*In* *vitro*: E-cadherin↓, Vimentin↑	*In* *vitro*: Mel Ibr cells	*In* *vitro*: 2% (v/v)	Suraeva et al. (2017)
Uveal Melanoma	Soybeans	Genistein	N/A	*In* *vitro*: VE-cadherin↓, PAS+↓ *In* *vivo*: CD34-/PAS+↓	*In* *vitro*: C918 cells *In* *vivo*: BALB/c-nude mice	*In* *vitro*: 25–200 μM *In* *vivo*: 75 mg/kg	Cong et al. (2009)
	Many fruits and green plants	Luteolin	PI3K/Akt	*In* *vitro*: p-PI3K↓, p-Akt↓, VEGF↓	*In* *vitro*: C918 cells	*In* *vitro*: 6.25–25 μM	Chen et al. (2022)
Choroidal Melanoma	Curcuma longa	Curcumin	EphA2/PI3K/MMPs	*In* *vivo*: CD31-/PAS+↓, EphA2↓, PI3K↓, MMP2/9↓	*In* *vivo*: C57BL/6J mice	*In* *vivo*: 100 mg/kg	Chen et al. (2011)
	Artemisia annua	Artesunate	HIF-1α/VEGF/PDGF	*In* *vitro*: HIF-1α↓, VEGF↓, PDGF↓	*In* *vitro*: OCM-1 cells	*In* *vitro*: 40 μM	Ma et al. (2024)
	Artemisia annua	Artesunate	Wnt/CaMKII	*In* *vitro*: PAS+↓, Wnt5a↓, p-CaMKII↓, HIF-1α↓, VE-cadherin↓, EphA2↓, VEGFA↓, VEGFR2↓, PDGFR↓	*In* *vitro*: OCM-1/C918 cells	*In* *vitro*: 2.5–1 μM (OCM-1 cells) 15–60 μM (C918 cells)	Geng et al. (2021)
Renal Cell Carcinoma	Curcuma longa	Curcumin	ETS-1/VE-Cadherin/MMP9	*In* *vitro*: ETS-1↓, VE-Cadherin↓, MMP9↓ *In* *vivo*: CD31-/PAS+↓, ETS-1↓, VE-Cadherin↓, MMP9↓	*In* *vitro*: 786-O/Caki-1 cells *In* *vivo*: BALB/c-nude mice	*In* *vitro*: 10 μM *In* *vivo*: 100–300 mg/kg	Chong et al. (2024)
Nasopharyngeal Carcinoma	Ginseng	Ginsenoside Rg3	N/A	*In* *vitro*: VEGF↓	*In* *vitro*: HNE-1 cells	*In* *vitro*: 50–200 μg/mL	Wang et al. (2010)
Gallbladder carcinoma	Mylabris	Norcantharidin	PI3K/MMPs/Ln5γ2	*In* *vitro*: PI3K↓, MMP2↓, MT1-MMP (MMP14)↓, Ln5γ2↓ *In* *vivo*: CD31-/PAS+↓, PI3K↓, MMP2↓, MT1-MMP (MMP14)↓, Ln5γ2↓	*In* *vitro*: GBC-SD cells *In* *vivo*: BALB/c-nude mice	*In* *vitro*: 28 μg/mL *In* *vivo*: 28 mg/kg	Zhang et al. (2014)
	Mylabris	Norcantharidin	N/A	*In* *vitro*: MMP2↓, MT1-MMP (MMP14)↓ *In* *vivo*: CD31-/PAS+↓, MMP2↓, MT1-MMP (MMP14)↓	*In* *vitro*: GBC-SD cells *In* *vivo*: BALB/c-nude mice	*In* *vitro*: 28 μg/mL *In* *vivo*: 28 mg/kg	Zhu et al. (2015)

Salvianolic Acid A (SAA) is a water-soluble compound primarily derived from Salvia miltiorrhiza, a plant of the Lamiaceae family. Jin et al. reported that in NCI-H1299 and A549 cells, SAA exerts anti-VM effects by interfering with the PI3K/Akt/mTOR signaling pathway. Specifically, it significantly reduces the protein expression levels of phosphorylated PI3K (p-PI3K), phosphorylated Akt (Ser473/Thr308) (p-Akt), and phosphorylated mTOR (Ser2448) (p-mTOR), while inhibiting the transcription and translation of VM-related factors such as VE-cadherin, EphA2, and MMP2 (Jin et al., 2021). Further functional assays demonstrated that SAA inhibits cell migration, invasion, and Matrigel-based tube formation in a dose-dependent manner. Notably, these inhibitory effects were fully reversed by pretreatment with the PI3K/Akt pathway activator SC79, thereby confirming the pivotal role of this signaling cascade.

Scoparasin B is a diterpenoid compound isolated from the marine-derived fungus Eutypella sp. F0219. Lin et al. demonstrated that in NCI-H1299 cells, Scoparasin B inhibits VM in lung cancer by targeting the VEGFA/VEGFR2 signaling axis. This effect is achieved through the downregulation of VM-related markers including VE-cadherin, N-cadherin, Vimentin, Snail, and MMP9, along with the upregulation of E-cadherin, thereby interfering with EMT and ECM remodeling processes associated with VM formation in lung cancer (Lin et al., 2023). Moreover, in the NCI-H1299 xenograft model, treatment with Scoparasin B significantly reduced the number of PAS-positive VM channels and the density of CD31-positive endothelial vessels. This was accompanied by an expansion of tumor necrotic areas. Additionally, VEGF-A expression levels were positively correlated with VM formation, underscoring the critical role of the VEGF-A/VEGFR2 signaling pathway as a central regulator in the interplay between angiogenesis and VM.

Bis(2,3,6-tribromo-4,5-dihydroxybenzyl)ether (BTDE) is a bromophenol compound derived from the marine red alga Symphyocladia latiuscula. Dong et al. demonstrated that BTDE significantly inhibits the formation of VM tubular structures in A549 cells, but has no significant effect on the expression of molecules such as VEGF, HIF-1α, β-catenin, or downstream signaling molecules in the AKT and ERK pathways. This suggests that BTDE may exert its anti-VM effects through alternative mechanisms (Dong et al., 2021).

Yi-Fei-Jie-Du-Tang (YFJDT) is a TCM formula originated in China, mainly composed of Hedyotis diffusa, Ophiopogon japonicus, Bombyx batryticatus, Sarcandra glabra, Glehniae Radix, Hairyvein Agrimonia Herb, Pseudostellariae Radix, Alismatis Rhizoma, Pleiones Pseudobulbus, and Radix Rnunculi Ternati (Wang et al., 2022b). Wang et al. demonstrated that YFJDT directly inhibits the expression of the VM-related protein VE-cadherin, reduces the formation of PAS^+^/CD31^-^ regions, and disrupts VM structures (Wang et al., 2025c). Additionally, YFJDT suppresses EMT by downregulating HIF-1α, decreasing the expression of EMT markers such as N-cadherin and Vimentin, while increasing E-cadherin expression. The inhibition of EMT directly weakens the ability of tumor cells to form VM. Interestingly, YFJDT relieves HIF-1α-mediated suppression of ferroptosis by upregulating ferroptosis-related factors ACSL4, TFR1, and 4-HNE, and downregulating GPX4 and SLC7A11, thereby inducing ferroptosis in A549 cells. Furthermore, the ferroptosis inducer Erastin also inhibits VM formation, demonstrating the significant inhibitory effect of ferroptosis on VM (Wang et al., 2025c). This also indirectly demonstrates that YFJDT achieves its anti-VM effect by promoting ferroptosis.

In summary, NPs and TCM can exert inhibitory effects on VM in lung cancer through multi-target and multi-pathway synergistic mechanisms. These mechanisms include regulating cellular phenotypic transitions, mediating cytoskeletal remodeling, suppressing signal transduction, promoting ECM remodeling, inhibiting EMT, and inducing ferroptosis. A common feature among these mechanisms is the targeting of the functional plasticity of tumor cells, thereby blocking their ability to acquire a non-endothelial-dependent blood supply and ultimately counteracting VM formation. Notably, the potential role of ferroptosis in VM inhibition has also garnered increasing attention. Although the underlying molecular mechanisms remain to be fully elucidated, preliminary studies have offered novel insights and opened new avenues for the development of VM-targeted therapeutic strategies.

### 3.2 Regulatory mechanisms of hepatocellular carcinoma

Polyphyllin I (PPI), a major active component of the TCM Paris polyphylla, has been shown to inhibit VM in HCC cell models such as SMMC7721 and PLC in a dose-dependent manner. PPI suppresses the PI3K/Akt signaling pathway by reducing Akt phosphorylation, thereby decreasing the nuclear translocation and promoter activity of the transcription factor Twist1. This inhibition blocks the binding of Twist1 to the VE-cadherin promoter, thereby repressing the transcriptional activation of VM-associated genes (including VE-cadherin, VEGFR1/2, MMP2, MMP9, Laminin 5, and vimentin), while simultaneously promoting the expression of the epithelial marker E-cadherin, ultimately contributing to the inhibition of VM formation in HCC (Xiao et al., 2018). In an *in vivo* xenograft model using nude mice, treatment with PPI significantly reduced the number of PAS-positive VM structures in tumor tissues and decreased the incidence of pulmonary metastases. Moreover, in tumor tissues from HCC patients treated with PPI, the density of VM structures (PAS^+^/CD31^-^) was markedly reduced, accompanied by the downregulation of VM-related markers (VE-cadherin, VEGFR1/2) and EMT markers (Twist1, Vimentin), as well as the upregulation of E-cadherin.

Celastrus orbiculatus extract (COE), a natural mixture of terpenoid compounds derived from the stem of Celastrus orbiculatus (family Celastraceae), has demonstrated significant activity against VM in HCC through multi-target and multi-pathway mechanisms (Jue et al., 2017b; Chu et al., 2021; Chen et al., 2024). In the regulation of the Notch1 signaling pathway, COE downregulates the mRNA and protein expression of the Notch1 receptor and its downstream target gene Hes1, while upregulating E-cadherin and concurrently downregulating Vimentin and VE-cadherin to effectively inhibit the EMT process, thereby reversing the endothelial-like phenotypic transition of tumor cells (Jue et al., 2017b). Under hypoxia-induced signaling regulation, hypoxic conditions induce the expression of HIF-1α, which directly or indirectly via c-Myc promotes the transcription of EphA2. This activation triggers downstream PI3K/FAK signaling pathways, leading to the upregulation of VE-cadherin expression and ultimately promoting VM formation in HCC. COE targets EphA2 and inhibits the expression of p-PI3K, phosphorylated FAK (p-FAK), VE-cadherin, MMP2/9, N-cadherin, and Vimentin, while reducing the nuclear translocation of Twist1 and upregulating E-cadherin expression. By blocking the HIF-1α/c-Myc–EphA2–PI3K/FAK/VE-cadherin signaling axis, COE effectively suppresses hypoxia-induced VM formation in HCC (Chu et al., 2021; Chen et al., 2024). *In vivo* experiments demonstrated that COE significantly decreases the immunohistochemical staining intensity of Notch1 and Hes1 in tumor tissues, downregulates the expression of EphA2, MMP2/9, VE-cadherin, Twist1, while upregulating E-cadherin expression, and reduces the number of CD31^-^/PAS^+^ and CD34^-^/PAS^+^ double-positive VM structures, further validating its inhibitory effect on VM formation in HCC (Jue et al., 2017b; Chu et al., 2021).

Curcumin, a natural active compound derived from Curcuma longa of the ginger family, significantly inhibits VM formation in the HCC SK-Hep-1 cell model by selectively suppressing the phosphorylation activation of the PI3K/AKT and STAT3 signaling pathways. Specifically, curcumin reduces the phosphorylation levels of AKT at Ser473 and STAT3 at Tyr705, thereby downregulating the transcription and activity of MMP9 and consequently blocking the VM capability of HCC cells (Chiablaem et al., 2014).

Incarvine C (IVC), an ester alkaloid derived from the TCM herb Incarvillea Sinensis, inhibits ROCK kinase activity in a concentration-dependent manner, reducing the phosphorylation level of MYPT-1 and disrupting the formation of actin stress fibers. Furthermore, IVC suppresses the transcriptional activity of VE-cadherin, EphA2, MMP2/9/14, PI3K, and LAMC2, as well as the protein expression of VE-cadherin, EphA2, and MMP2/9, thereby blocking tumor cell adhesion, ECM degradation, and the maintenance of vascular-like structures in HCC (Zhang et al., 2015).

Melittin, a natural active component of bee venom, inhibits VM formation in the CoCl_2_-induced hypoxia model of SMMC-7721 cells by reducing HIF-1α protein stability, decreasing the expression of VEGF and MMP2/9, blocking Akt phosphorylation, suppressing HIF-1α nuclear translocation, restoring E-cadherin expression, and downregulating N-cadherin and Vimentin expression. *In vivo* experiments further validated its multi-target synergistic mechanism of “inhibiting the HIF-1α/Akt axis, reversing EMT, and downregulating VM-associated factors” by demonstrating reduced HIF-1α expression and VM structures with CD34^-^/PAS^+^ staining in xenograft tumors of nude mice treated with Melittin (Chen et al., 2019).

Daurisoline (DS), an isoquinoline alkaloid derived from the TCM plant Rhizoma Menispermi, inhibits VM in HCC by directly suppressing the GTP-binding activity of RhoA, downregulating the expression of ROCK2, and inhibiting ROCK2-mediated phosphorylation of MYPT-1 and MLC-2. This upstream inhibition is further transmitted through downstream signaling cascades, leading to decreased phosphorylation levels of AKT (Ser473/Thr308), ERK1/2, and p38. As a result, the expression of VEGFR-2, VE-cadherin, and MMP2/9 is reduced. *In vivo*, DS significantly decreases CD34^-^/PAS^+^ VM structures in HCC mouse models, thereby effectively suppressing the development of VM (Zhang et al., 2021c).

Myricetin, a natural flavonoid primarily found in various fruits and vegetables, inhibits VM in HCC by specifically binding to the active pocket of protease-activated receptor 1 (PAR1) at residues Leu258 and Thr261 to block PAR1 activation. This leads to the downregulation of HIF-1α and Twist1, upregulation of E-cadherin, and suppression of VE-cadherin and VEGFR1/2 expression. *In vivo*, myricetin reduces the number of CD31^-^/PAS^+^ VM structures, ultimately inhibiting the formation of VM channels in HCC (Wang et al., 2022a).

Withaferin A (WA) is a natural steroidal lactone derived from Withania somnifera. Studies have shown that in HepG2 and SNU449 cells, WA activates the Keap1/Nrf2 signaling pathway by increasing the expression of Keap1 and decreasing the expression of Nrf2 protein. This activation suppresses the expression of VE-cadherin, N-cadherin, SLC7A11, and GSH, while upregulating E-cadherin, reactive oxygen species (ROS), and malondialdehyde (MDA), thereby reversing EMT and inducing ferroptosis to exert its inhibitory effects on VM in HCC.

Flavokawain A (FKA) is a natural chalcone compound isolated from the medicinal herb *Chloranthus henryi*. Studies have shown that in HCC cells such as HepG2 and SMMC-7721, FKA directly binds to CXCL12, blocking the CXCL12/CXCR4 signaling pathway and inhibiting PI3K/Akt phosphorylation. This results in the downregulation of NF-κB and HIF-1α, reduced expression of Twist1, reversal of EMT, suppression of VE-cadherin, vimentin, and Snail1, and upregulation of E-cadherin. *In vivo* experiments further demonstrated that FKA significantly reduces the number of PAS^+^/CD31^-^ VM structures, thereby exerting its anti-VM effects in HCC (Xiao et al., 2023).

Isoxanthohumol, a flavonoid compound isolated from the fungus Trichoderma harzianum, inhibits VM in HCC by targeting the TGF-β/Smad signaling pathway. Specifically, it directly blocks the binding ability of activated Smad2/3 transcription factors to the high-affinity CAGA box elements in DNA, rather than suppressing the expression of phosphorylated Smad2/3 proteins, thereby inhibiting TGF-β-induced transcriptional activation. This subsequently leads to the downregulation of MMP2, PDGF, and AngptL4 expression, ultimately suppressing VM formation in HCC (Serwe et al., 2012).

Biejiajian pill, a TCM formula, has been reported to suppress malignant biological processes in HCC, including migration, invasion, and the promotion of apoptosis (He et al., 2013; Wen et al., 2016). In a three-dimensional (3D) culture model of HepG2 cells, Biejiajian pill suppresses the expression of key molecules in the RhoA/ROCK signaling pathway, namely, RhoA and ROCK1, and reduces the levels of VE-cadherin and PI3K in the culture supernatant. These effects disrupt the interaction between tumor cells and the ECM, thereby impairing the microenvironmental remodeling required for VM in HCC (An et al., 2018).

Compound Taxus Capsule (CTC), a TCM formulation consisting of Taxus chinensis, red ginseng, and Glycyrrhiza uralensis, exhibits antitumor activity and is commonly used as an adjuvant treatment for advanced lung cancer (Li et al., 2021; Wang et al., 2024b). In HCC, CTC exerts anti-VM effects by suppressing the expression of VEGFA and MMP9, inhibiting the EMT process through downregulation of N-cadherin and Vimentin, and upregulating the protein expression of E-cadherin (Gao et al., 2024).

Overall, current studies on NPs and TCM for the treatment of VM in HCC have primarily focused on multiple mechanisms, including the disruption of CAMs expression, inhibition of EMT, reduction of ECM remodeling, suppression of hypoxia-mediated regulation, and blockade of key signaling pathways. Additionally, a relatively large number of studies have concentrated on HCC, which may be attributed to the unique biological characteristics of this cancer type. As the largest solid organ in the human body, the liver possesses a unique dual blood supply system, with both the portal vein and hepatic artery providing abundant blood flow, a feature that makes the liver a highly vascularized organ (Torres Rojas and Lorente, 2023). During the malignant transformation of normal hepatocytes, tumor cells aberrantly recruit branches of the hepatic artery, accompanied by the loss of fenestrations in the hepatic sinusoidal endothelium and the formation of continuous basement membranes resembling capillary-like structures, which hinder substance exchange and lead to arterialization of blood vessels and capillarization of hepatic sinusoids (Pinto et al., 2023; Morse et al., 2019). These abnormal changes promote the activation of the HIF-1α and VEGF/VEGFR signaling pathways, resulting in severe hypoxia within the tumor microenvironment, which in turn enhances tumor cell plasticity and the formation of vascular-like channels (Pinto et al., 2023; Morse et al., 2019; Motoo et al., 1993). However, the precise incidence of VM in various tumors has not been reported in detail and requires further research to validate this hypothesis.

### 3.3 Regulatory mechanisms of breast cancer

Grape seed proanthocyanidins, natural polyphenolic compounds extracted from grape seeds, inhibit VM formation in triple-negative breast cancer (TNBC) HCC1937 cells by downregulating Twist1 protein expression, decreasing VE-cadherin levels, and upregulating epithelial markers such as E-cadherin, thereby reversing the EMT process (Luan et al., 2015).

Phycocyanin, a natural active compound extracted from Arthrospira platensis, inhibits VM formation in TNBC cells by suppressing ERK1/2 phosphorylation in the MAPK/ERK pathway, downregulating COX-2 expression, and reducing PGE2 production, which in turn decreases the transcriptional levels of VEGFR2 and MMP9 (Ravi et al., 2015).

Brucine, an indole alkaloid extracted from seeds of Loganiaceae plants, inhibits VM formation in TNBC MDA-MB-231 cells by targeting and downregulating the expression of pro-angiogenic factors EphA2 and MMP2/9. This action blocks the initiation of VM channels and weakens the degradation capacity of the ECM. Additionally, brucine inhibits the directional arrangement of F-actin and the formation of microtubule structures, thereby disrupting the integrity of the tumor cell cytoskeleton (Xu et al., 2019).

Thymoquinone (TQ), a natural phytochemical derived from the seeds of Nigella sativa Linn, has been shown to inhibit VM in breast cancer stem cells by targeting the PI3K/Akt and Wnt3a signaling pathways. TQ suppresses the expression of key mesenchymal-to-endothelial transition (MEndT) markers, including VE-cadherin, MMP2, and MMP9, attenuates stemness properties-as indicated by a reduced proportion of the CD24^−^ subpopulation-and inhibits Rhodamine 123 efflux mediated by ATP-binding cassette transporters. Moreover, TQ antagonizes the cellular responsiveness to angiogenic factors such as VEGF, EGF, and FGF, thereby effectively blocking VM channel formation and synergistically reversing VM-associated drug resistance (Haiaty et al., 2021).

Triptonide, a natural small-molecule compound extracted from the TCM herb Tripterygium wilfordii, inhibits VM in TNBC by inducing the lysosomal degradation of Twist1 and Notch1 proteins, suppressing NF-κB phosphorylation, and downregulating the expression of VE-cadherin, VEGFR2, and N-cadherin (Zhang et al., 2021a). These combined effects contribute to the effective inhibition of VM progression in TNBC.

Sinomenine, a natural isoquinoline alkaloid extracted from Sinomenium acutum, inhibits VM formation and migration in breast cancer stem-like side population cells. Mechanistically, sinomenine upregulates miR-340-5p, which in turn suppresses the expression of SIAH2, thereby blocking the HIF-1α signaling pathway. This leads to the downregulation of key VM-associated factors such as VE-cadherin, MMP9, and EphA2, while simultaneously increasing E-cadherin expression and reducing levels of mesenchymal markers N-cadherin and Snail (Song et al., 2022).

Curcumin suppresses VM in TNBC cells (MBCDF-T and HCC1806) by inhibiting the activity of the PI3K/Akt and MAPK signaling pathways. It downregulates the expression of multiple phosphorylated proteins, including p-Akt, p-CREB, p-GSK3-α/β, p-ERK1/2, p53, p38, p-JNK, p-Hsp27, and p70S6K. Without altering cell morphology, curcumin reduces the branching and mesh formation of tubular networks, thereby disrupting VM structures through the impairment of cell–cell adhesion molecules. Notably, its anti-VM effect is further enhanced when used in combination with the endogenous vitamin D metabolite, calcitriol (Li et al., 2022a).

Epigallocatechin gallate (EGCG), a natural polyphenolic compound extracted from the leaves of green tea, exerts its anti-VM effects primarily through inhibition of the STAT3 signaling pathway. Specifically, EGCG significantly downregulates the phosphorylation of STAT3 at Tyr705, blocks its nuclear translocation, and suppresses the expression of Fibronectin, a key mesenchymal marker. Furthermore, EGCG attenuates the adipokine-mediated phosphorylation cascade of the JAK/STAT3 pathway, thereby synergistically counteracting the adipose microenvironment-induced, STAT3-dependent drug-resistant phenotype. These mechanisms collectively contribute to the inhibition of migration, invasion, and VM formation in TNBC cells (Gonzalez Suarez et al., 2021).

Xian-ling-lian-xia-fang (XLLXF), a traditional Chinese medicinal formula, contains multiple bioactive components such as quercetin, kaempferol, stigmasterol, and β-sitosterol. XLLXF inhibits VM formation in TNBC MDA-MB-231 cells through a multi-component, multi-target synergistic mechanism. It suppresses the VEGF/MMP signaling pathway by downregulating the expression of VEGFA, MMP2, and MMP9, while upregulating tissue inhibitors of metalloproteinases (TIMP-1 and TIMP-3) to reduce ECM degradation. In addition, XLLXF reverses the EMT by increasing E-cadherin expression and decreasing the levels of Vimentin, Twist1, and VE-cadherin, thus preventing tumor cells from acquiring an endothelial-like phenotype. Furthermore, XLLXF disrupts the organization of the F-actin cytoskeleton, inhibiting pseudopodia formation and migration capacity of tumor cells (Li et al., 2022a).

In summary, NPs and TCM have demonstrated significant potential in inhibiting VM in breast cancer, particularly in the highly aggressive TNBC subtype. These agents exert multi-targeted and multi-pathway synergistic effects by interfering with key processes of VM formation, including regulation of EMT, inhibition of signaling pathways, remodeling of the ECM, disruption of the cytoskeleton, and targeting of CSCs, thereby offering new therapeutic possibilities for breast cancer. However, the incidence and clinical relevance of VM in different breast cancer subtypes-such as hormone receptor-positive and HER2-positive tumors-remain unclear and warrant further investigation to inform subtype-specific therapeutic strategies.

### 3.4 Regulatory mechanisms of glioblastoma

Myricetin, a natural flavonoid compound, directly targets and binds to VE-cadherin and the catalytic subunits of PI3K (p110α, p110β, and p110δ), leading to reduced PDK1 activity. This results in the inhibition of phosphorylation of Akt, c-Jun, ROCK2, Paxillin, and Cortactin, disrupting focal adhesion maturation and actomyosin contractility, thereby significantly suppressing VM tubular network formation of glioblastoma U-87 MG cells in a dose-dependent manner *in vitro* (Zhao et al., 2018).

Curcumin dose-dependently downregulates the expression of EphA2, PI3K, and MMP2 to block the EphA2/PI3K/MMP-2 signaling pathway, thereby suppressing ECM remodeling as well as glioblastoma cell migration and invasion, ultimately disrupting VM formation in glioblastoma (Liang et al., 2014).

Celastrol, a small-molecule compound extracted from Tripterygium wilfordii, inhibits the activity of the PI3K/Akt/mTOR signaling pathway by reducing the expression of phosphorylated PI3K (p-PI3K), phosphorylated Akt (p-Akt), and phosphorylated mTOR (p-mTOR). It also downregulates the levels of EphA2, VE-cadherin, and VEGFR2, suppresses HIF-1α expression, and further blocks its transcriptional activation of VM-related genes. Additionally, Celastrol can decrease CD31^-^/PAS^+^ expression *in vivo*, thereby disrupting VM progression in glioblastoma (Zhu et al., 2020).

N6-isopentenyladenosine (iPA) is a modified nucleoside generated through natural metabolic pathways. It inhibits Src kinase phosphorylation, thereby reducing Src activity and subsequently decreasing phosphorylation of p120-catenin. This disruption impairs the interaction of p120-catenin with VE-cadherin and E-cadherin, leading to the disassembly of adherens junctions (AJs). Notably, E-cadherin functions as a scaffold protein for AJs in this process, and it is co-expressed with VE-cadherin in glioma cells, forming heterotypic junctions that jointly maintain tubular structures. Additionally, iPA suppresses RhoA GTPase activity by blocking the translocation of RhoA from the cytoplasm to the plasma membrane, reducing the active GTP-bound form of RhoA. This inhibition of the RhoA/ROCK pathway-mediated actin stress fiber formation ultimately disrupts VM network construction in glioblastoma (Pagano et al., 2021).

Juglone, a natural naphthoquinone compound derived from walnut trees, inhibits VM formation in glioblastoma by targeting the RNA-binding protein HuR. It suppresses the interaction between HuR and the AU-rich element (ARE) region of VEGFA mRNA, thereby reducing the expression and secretion of phosphorylated VEGFA (p-VEGFA). This leads to the downregulation of p-VEGFR2 and p-Akt expression and the blockade of VEGFR2/AKT signaling pathway activation. In addition, juglone inhibits the transcription factor SNAIL, resulting in the upregulation of E-cadherin and the downregulation of Vimentin and VE-cadherin (Luo et al., 2024). It also decreases CD31^-^/PAS^+^ expression *in vivo*. Collectively, these effects highlight the potential of juglone as a novel HuR inhibitor for targeting VM in glioblastoma therapy.

Stigmasterol, a sterol compound found in various TCM, inhibits VM formation in glioblastoma by interfering with lipid metabolic reprogramming. It directly binds to key lipid metabolism targets, including fatty acid-binding protein 5 (FABP5), which promotes lipid droplet accumulation and tumor invasion, and α-1B adrenergic receptor (ADRA1B), which regulates fatty acid synthase activity. Stigmasterol significantly suppresses the expression of both targets, thereby reducing intracellular levels of free fatty acids (FFAs) and total cholesterol (T-CHO) in glioma cells (Wei et al., 2024). This inhibition of lipid metabolic reprogramming disrupts lipid raft integrity and energy supply in tumor cell membranes, ultimately impairing VM formation in glioblastoma (Lu et al., 2023; Seo et al., 2020; Liu et al., 2012a).

In addition, studies have shown that Ruta graveolens water extract (RGWE) significantly inhibits VM structure formation in glioblastoma cell lines U87, U251, C6, and patient-derived GBM cancer stem cells (CSCs), with inhibition rates of 65%, 79%, 60%, and 50%, respectively, and also markedly reduces VEGF gene expression levels (Camerino et al., 2024). RGWE also markedly reduces VEGF gene expression levels. However, its underlying mechanisms have not been thoroughly investigated. It is speculated that RGWE may exert its effects by blocking the MEK–ERK1/2 signaling axis, thereby downregulating VEGFA and nestin gene expression (Camerino et al., 2024; Gentile et al., 2018). Notably, the anti-VM effect of RGWE results from the synergistic action of multiple components, as its major constituent rutin exhibits no significant activity on the relevant mechanisms at an equivalent concentration.

The above studies systematically elucidate that various NPs effectively inhibit VM in glioblastoma by targeting key molecules involved in VM formation, such as VE-cadherin and EphA2, as well as modulating multiple signaling pathways including PI3K/Akt/mTOR and EphA2/PI3K/MMP-2. These agents disrupt cell junctions, cytoskeletal dynamics, ECM remodeling, hypoxia responses, lipid metabolism, and the expression of critical pro-VM-related genes. Collectively, these findings provide a crucial molecular basis and potential candidate compounds for the development of therapeutic strategies based on NPs against glioblastoma VM.

### 3.5 Regulatory mechanisms of gastric cancer

Dehydroefiusol (DHE), a natural phenanthrene compound isolated from the TCM herb Juncus effusus, dose-dependently suppresses VE-cadherin promoter activity and downregulates the expression of β-catenin, MMP2, and p-ERK. By disrupting tumor cell adhesion junctions and inhibiting ECM degradation, DHE effectively impedes VM formation in Gastric cancer (GC) (Liu et al., 2015).

Luteolin, a natural compound found in various fruits and plants, targets the Notch1/VEGF signaling axis by downregulating Notch1 expression and inhibiting VEGF secretion. This action blocks the ability of GC cells to form VM tubular structures and concurrently suppresses angiogenesis by endothelial cells within the tumor microenvironment.

Crocetin, a natural active compound extracted from saffron stigma, exhibits multiple antitumor effects (Moradzadeh et al., 2018). It targets the Sonic hedgehog (SHH) signaling pathway by inhibiting SHH ligand secretion, disrupting the cytoskeleton, and downregulating the expression of PTCH2, SUFU, and Gli1, thereby directly blocking the ability of GC cells to form VM (Zang et al., 2021).

Dihydroartemisinin (DHA), a natural product extracted from the traditional medicinal herb Artemisia annua, inhibits VM formation in GC cells by suppressing FGF2 transcription and reducing the binding of FGF2 to FGFR1. This leads to inhibition of downstream Ras/Raf/MEK/MAPK and PI3K/AKT/mTOR signaling pathways, resulting in the downregulation of Twist1, MMP2, Vimentin, VE-cadherin, MMP9, N-cadherin, and CD34^-^/PAS^+^ expression, thereby ultimately blocking VM tubular structure formation (Wang et al., 2024a).

Xiaotan Sanjie Recipe (XTSJR), a traditional Chinese medicinal formula derived from China, mainly consists of Arisaema heterophyllum Blume, Pinellia ternata, Buthus martensii Karsch, Scolopendra subspinipes mutilans, Gekko swinhonis, and Duchesnea indica. Its molecular mechanism for inhibiting VM in GC involves downregulating the protein expression of MMP2 and MMP9, reducing the number of CD34^-^/PAS^+^ structures, and impairing ECM degradation. This disruption prevents tumor cells from deforming and interacting with the ECM to form vascular-like lumen structures, ultimately suppressing VM in GC (Zhou et al., 2011).

The aforementioned NPs and TCM inhibit VM formation in GC by targeting MMPs, CAMs, pro-angiogenic factors, and key signaling pathways. These agents disrupt ECM degradation, cell junction integrity, cytoskeletal dynamics, and the EMT, thereby effectively suppressing VM formation. Notably, the herbal formula XTSJR and DHA demonstrate broad-spectrum regulatory capabilities. In particular, in-depth mechanistic studies on DHA have unveiled the crosstalk between EMT and VM, offering a strong foundation for combined targeted therapeutic strategies against tumor VM.

### 3.6 Regulatory mechanisms of prostate cancer

Epigallocatechin-3-gallate (EGCG), the most abundant polyphenolic compound in green tea, has been shown to dose-dependently downregulate the expression of the transcription factor Twist and inhibit its nuclear translocation. This suppression leads to reduced expression of downstream targets including VE-cadherin, N-cadherin, and Vimentin, as well as decreased phosphorylation and total levels of AKT. In summary, these effects indicate that EGCG inhibits VM formation in prostate cancer (PCa) PC-3 cells by targeting the Twist/VE-cadherin/AKT signaling axis (Yeo et al., 2020).

Resveratrol (RES), a natural compound found in grapes, berries, and peanuts, has been shown to dose-dependently inhibit serum-induced phosphorylation of EphA2 and suppress the nuclear expression of Twist. This results in the downregulation of VE-cadherin, p-AKT, MMP2, and laminin γ2 (LAMC2), thereby forming an EphA2/Twist–VE-cadherin/AKT/MMP2/LAMC2 signaling cascade that ultimately inhibits VM formation in PCa PC-3 cells (Han et al., 2022).

Studies have shown that kaempferol, a natural flavonol compound found in various fruits and vegetables, significantly inhibits VM formation and invasive ability of androgen receptor-negative PCa cells in a dose-dependent manner (Da et al., 2019). However, its underlying molecular mechanisms remain insufficiently explored. This effect may be achieved by inhibiting the VEGF-mediated ERK-NFκB-cMyc-p21 signaling axis and EMT-related factors, thereby interfering with the functional expression of key VM proteins VE-cadherin and EphA2, ultimately blocking the VM process in PCa cells (Luo et al., 2012; Wang et al., 2018; Yeo et al., 2019).

Luteolin and quercetin, as natural dietary flavonoids, have been reported to inhibit the activation of the JNK signaling pathway by blocking JNK phosphorylation and subsequent JUN activation. They also reduce the expression of MMP9, thereby weakening ECM degradation and suppressing PCa cell migration and the formation of a microenvironment conducive to VM. Additionally, luteolin and quercetin decrease the expression of CSCs markers Nanog, Sox2, CD44, and ABCG2, impairing the self-renewal capacity of CSCs and ultimately inhibiting VM in PCa (Tsai et al., 2016).

Qilan Capsules, a traditional herbal formula originating from China, primarily consist of Astragalus membranaceus var. mongholicus, Gynostemma pentaphyllum, Trigonella foenum-graecum, Scarabaeus spp., and Smilax glabra. Qilan Capsules have been found to inhibit VM tube formation in PC-3 cells of PCa by downregulating HIF-1α expression, suppressing VEGFα transcriptional activation, and reducing MMP1 activity, thus targeting the HIF-1α/VEGFα/MMP-1 signaling axis (Yu et al., 2018).

In summary, current studies indicate that NPs and TCM primarily inhibit VM formation in PCa by targeting the expression of key VM-related molecules such as VE-cadherin, EphA2, Twist, and MMPs, and suppressing the activation of signaling pathways including JNK/JUN/MMP9 and HIF-1α/VEGFα/MMP-1. These actions disrupt cell migration and invasion, ECM remodeling, hypoxic responses, CSCs self-renewal capacity, as well as the activity and expression of critical pro-VM transcription factors. Through this multi-targeted interference, NPs and TCM hold promise as novel therapeutic strategies for targeting VM in PCa. Notably, current studies primarily utilize androgen-independent PC-3 cell lines, suggesting that these anti-VM effects may be particularly relevant in advanced, hormone-refractory PCa. This also raises an important question: whether these findings can be extended to androgen-dependent PCa models remains to be further investigated.

### 3.7 Regulatory mechanisms of pancreatic cancer

Ginsenoside Rg3, a minor tetracyclic triterpenoid saponin extracted from ginseng, inhibits VM in pancreatic cancer (PC) cells by targeting the VE-cadherin/EphA2/MMP2/MMP9 signaling axis. Specifically, Ginsenoside Rg3 blocks the nuclear translocation of key EphA2-associated signaling molecules, suppresses the enzymatic activity of MMP2 and MMP9 to prevent ECM degradation, and disrupts VE-cadherin-mediated intercellular adhesion (Guo et al., 2014).

Furthermore, Ginsenoside Rg3 significantly upregulates the expression of miR-204 in exosomes derived from PC cells (SW-1990 and PCI-35). miR-204 directly binds to the 3′UTR of Dishevelled 3 (DVL3) mRNA, leading to downregulation of DVL3 protein expression and subsequent inhibition of Wnt/β-catenin and PI3K/AKT pathway activities. This process disrupts CSCs properties, as evidenced by decreased levels of stemness markers OCT4, SOX2, and Nanog. Moreover, Ginsenoside Rg3 upregulates E-cadherin and downregulates N-cadherin and Vimentin expression, thereby suppressing the formation of CD31^-^/PAS^+^ tubular structures in xenograft tumors (Cai et al., 2024).

Under hypoxia-induced tumor microenvironment, activated HIF-1α promotes the expression of leucyl-tRNA synthetase 1 (LARS1) in exosomes derived from PC cells. These exosomes are subsequently taken up by recipient PC cells, leading to activation of the mTOR signaling pathway and facilitating VM formation. Ginsenoside Rg3 has been shown to dose-dependently downregulate the expression of HIF-1α and LARS1, block the nuclear translocation of HIF-1α, and inhibit mTOR phosphorylation and the expression of its downstream targets VEGF and VE-cadherin, thereby suppressing VM by targeting the HIF-1α/LARS1/mTOR signaling axis in PC cells (Zhao et al., 2025).

In addition, studies have shown that triptonide suppresses the expression of the VM-associated gene VE-cadherin and the pro-migratory gene CXCL2 in a dose-dependent manner. It inhibits their transcription by suppressing the promoter activities of both genes, thereby reducing the migratory and invasive capabilities of PC cells. These findings suggest that triptonide exerts anti-VM effects in PC by targeting the VE-cadherin/CXCL2 signaling axis (Han et al., 2018).

The above results demonstrate that Ginsenoside Rg3 primarily inhibits VM formation in PC through a comprehensive triple mechanism involving disruption of cell adhesion, reversal of stemness properties, and blockade of hypoxia responses. In contrast, triptonide targets the VE-cadherin/CXCL2 axis to suppress migration and invasion. Both compounds converge on the core target VE-cadherin, while expanding anti-VM strategies by modulating exosomal miRNA networks and the chemokine CXCL2, respectively. These multidimensional mechanisms provide a robust molecular basis for the development of NPs targeting PC VM.

### 3.8 Regulatory mechanisms of osteosarcoma

Coptisine, a major active alkaloid extracted from Coptidis rhizoma, dose-dependently downregulates the expression of genes and proteins including VE-cadherin, integrin β_3_, VEGFA, MMP2, integrin α5, and integrin β5, thereby disrupting intercellular adhesion and inhibiting cell migration and invasion. Meanwhile, coptisine specifically blocks the phosphorylation of STAT3 at the Tyr705 residue, synergistically suppressing capillary-like network formation driven by osteosarcoma cells, ultimately effectively inhibiting VM in osteosarcoma MG63 cells (Yu et al., 2014).

Paris polyphylla ethanol extract (PPEE) is a natural extract derived from the TCM herb Paris polyphylla. PPEE inhibits VM formation in osteosarcoma both *in vitro* and *in vivo* by downregulating the expression of FAK and migration-inducing gene 7 (MIG-7), suppressing the activities of MMP2 and MMP9, and reducing the number of CD31^-^/PAS^+^ channels *in vivo* (Yao et al., 2017).

Gracilistylacid C and impressic acid are triterpenoids isolated from Acanthopanax senticosus. They exhibit anti-VM effects by significantly inhibiting migration and tube formation of osteosarcoma 143B cells, independent of cytotoxicity. However, the specific biological mechanisms underlying their anti-VM activity remain unexplored and warrant further investigation (Li et al., 2022b).

Rhizoma Paridis is a TCM herb known for its heat-clearing and detoxifying properties. Rhizoma Paridis saponins (RPS) are the primary saponin components extracted from Rhizoma Paridis. Studies have shown that RPS dose-dependently upregulates the expression of miR-520d-3p and promotes its binding to MIG-7 mRNA, thereby inhibiting the expression of downstream MIG-7, p-PI3K, p-AKT, and p-mTOR. This further disrupts F-actin remodeling and suppresses the activities of MMP2, MMP14, and the cleavage of Ln5γ2, demonstrating the inhibition of VM in osteosarcoma cells by targeting the miR-520d-3p/MIG-7/PI3K/MMPs/Ln5γ2 signaling axis (Yao et al., 2022).

Based on the aforementioned findings, we identified that the core mechanisms by which NPs and TCM target VM in osteosarcoma focus on the regulation of adhesion and migration systems, ECM degradation networks, and key signaling hubs. These studies collectively highlight VE-cadherin, integrins, MIG-7, and MMPs as critical targets in osteosarcoma VM. Importantly, the dual-pathway synergistic inhibition by coptisine and the miRNA-MIG-7 cascade regulation by RPS offer multidimensional strategies for anti-VM therapies.

### 3.9 Regulatory mechanisms of colorectal cancer

Evodiamine (Evo) is a quinazoline alkaloid isolated from Evodia rugulosa that exhibits a range of pharmacological activities, including antitumor effects. Studies have shown that Evo downregulates the expression of HIF-1α in a dose-dependent manner and blocks its nuclear translocation, thereby suppressing the downstream expression of VE-cadherin, VEGF, MMP2, and MMP9. This leads to a reduction in CD31^-^/PAS^+^ double-stained VM channels *in vivo*, indicating that Evo inhibits VM in colorectal cancer (CRC) by targeting the HIF-1α/VE-cadherin/VEGF/MMPs signaling axis (Zeng et al., 2021).

Matrine, the primary alkaloid extracted from the TCM Sophora flavescens, downregulates the expression of Claudin-9 (Cldn9) in a dose- and time-dependent manner. This suppression further inhibits the expression of N-cadherin, MMP2, and MMP9 involved in EMT, while upregulating E-cadherin expression. Additionally, matrine inhibits the phosphorylation of JNK and ERK in the MAPK signaling pathway, thereby suppressing VM formation in CT-26 CRC cells (Du et al., 2023).

Yi Ai Fang (YAF), a traditional Chinese herbal formula composed of Astragali Radix, Macrocephalae Rhizoma, Actinidia arguta, and Curcuma zedoaria, has been shown to downregulate the expression and block the activity of HIF-1α in a dose-dependent manner. This inhibition leads to decreased expression of the mesenchymal marker Vimentin and increased expression of epithelial markers E-cadherin and Claudin-4, thereby reducing the plasticity and migratory ability required for VM formation in HCT-116 cells, resulting in YAF effectively suppressing the formation of VM tubular network structures in CRC (Hou et al., 2016).

Astragalus Atractylodes Mixture (AAM) is a traditional herbal formula consisting of six herbs, including Astragalus membranaceus Fisch, Atractylodes macrocephala Koidz, Actinidia arguta, Curcuma aromatica, Benincasa hispida and Ficus pumila L. Studies have shown that AAM reduces reactive oxygen species (ROS) levels in CRC cells under hypoxic conditions, thereby alleviating ROS-mediated inhibition of prolyl hydroxylases (PHDs). This leads to decreased stability and nuclear translocation of HIF-1α, resulting in downregulation of MMP2 expression. Consequently, AAM effectively suppresses VM formation in CRC HCT-116 and LoVo cells by targeting the ROS/HIF-1α/MMP2 signaling pathway (Zong et al., 2020).

The above findings indicate that NPs and TCM primarily inhibit VM in CRC by interrupting hypoxia-driven VM initiation signals within the tumor microenvironment, reversing the EMT phenotype, suppressing tumor cell plasticity and channel formation capacity, and blocking ECM remodeling. This ultimately inhibits the assembly of VM tubular structures and offers a multi-target synergistic strategy to overcome resistance to conventional anti-angiogenic therapies. Current research predominantly focuses on cell lines such as HCT-116, CT-26, and LoVo, indicating the necessity to validate the universality of these mechanisms across a broader spectrum of CRC models. However, the mechanisms underlying VM inhibition in CRC have rarely addressed the role of CSCs properties. Future studies could explore the involvement of stem cell-related markers in VM to further elucidate its molecular basis.

### 3.10 Regulatory mechanisms of ovarian cancer and choriocarcinoma

Gallated catechins, a group of dietary polyphenols found in green tea and other sources, inhibit the binding of TGF-β to its receptors TGF-β RI/II, thereby blocking receptor oligomerization and kinase activity. This leads to reduced phosphorylation of Smad3 and p38, downregulation of Snail, Slug, fibronectin, and vimentin expression, as well as decreased secretion of MMP2. As a result, the EMT process is interrupted and ECM degradation is suppressed, ultimately disrupting the formation of VM-related 3D capillary-like structures in OC cells (Sicard et al., 2021).

Cinobufagin, a water-soluble active compound extracted from the skin of *Bufo gargarizans*, suppresses VM in OC by downregulating FOXS1 expression and inhibiting the activation of the CCL2/CCR2 signaling pathway. This blockade prevents the polarization of TAMs toward the M2 phenotype-evidenced by the upregulation of CD86^+^, iNOS, and IL-12, and the downregulation of CD206^+^, Arg-1, IL-10, and TGF-β. Consequently, the expression of key VM effector proteins LAMC2, MMP9, and MMP14 is reduced, and the formation of PAS^+^/CD31^-^ tubular structures is suppressed *in vivo*, thereby exerting an anti-VM effect against OC (Wang et al., 2025a).

Moreover, it is worth noting that while numerous NPs and TCM have demonstrated inhibitory effects on tumor VM, studies have also begun to explore the molecular mechanisms by which certain specific NPs may promote tumor VM.

Forskolin, a diterpenoid compound extracted from the root of Coleus forskohlii (a plant in the Lamiaceae family), acts as an activator of the cAMP signaling pathway and has been shown to promote VM in choriocarcinoma JEG-3 and JAR cells. Specifically, forskolin induces syncytiolization of trophoblast cells and activates the Notch-1 signaling pathway, leading to the production of the Notch intracellular domain (NICD) and increased expression of HES1. This cascade subsequently triggers the EMT process, characterized by upregulation of N-cadherin, Vimentin, and ZEB1 and downregulation of E-cadherin and CK19. These changes suppress the expression of VE-cadherin, EphA2, and MMP2/9, while promoting the formation of CD31^-^/PAS^+^ vascular-like channels *in vivo*, thereby facilitating VM in choriocarcinoma cells via the cAMP/Notch-1 signaling axis (Xue et al., 2020).

Current studies on NPs inhibiting VM formation in OC suggest that their anti-VM effects are mainly exerted by suppressing EMT, promoting ECM degradation, and regulating the tumor microenvironment through the inhibition of macrophage polarization. In contrast, forskolin has been reported to promote VM formation in choriocarcinoma by activating the Notch-1 signaling pathway via cAMP, representing the only known case in which single natural product facilitates tumor VM formation. This finding underscores the potential for signaling crosstalk induced by NPs to generate unexpected effects, highlighting the bidirectional nature of their role in VM regulation. Such insights offer valuable guidance for future studies aimed at elucidating the complex relationship between NPs and tumor VM.

### 3.11 Regulatory mechanisms of esophageal squamous cell carcinoma

Folic acid, a water-soluble B vitamin abundant in leafy green vegetables and animal liver, specifically inhibits the expression of the EphA2 gene and protein in esophageal squamous cell carcinoma (ESCC) ECA-109 cells, thereby suppressing the formation of VM structures. However, it does not significantly affect VM-associated factors such as MMP2/9, VE-cadherin, or Ln-5γ2 (Xu et al., 2022).

Aidi injection is an injectable formulation extracted from natural medicines such as Panax ginseng, Astragalus membranaceus, Eleutherococcus senticosus, and Mylabris. Its main active components primarily include astragalosides, ginsenosides, and eleutherosides. Studies have shown that Aidi injection dose-dependently inhibits the expression of VEGFA and suppresses VM in ESCC cells by blocking EMT through upregulating E-cadherin and downregulating N-cadherin and vimentin (Shi et al., 2018).

Modified Tongyou decoction (MTD) is a traditional Chinese herbal formula composed of Areca catechu, Carthamus tinctorius, Cimicifuga heracleifolia, Prunus persica, Scutellaria barbata, and Hedyotis diffusa. Studies have demonstrated that MTD is capable of inhibiting NF-κB activation and HIF-1α accumulation, downregulating the expression of MMP2/9, VE-cadherin, Snail, Vimentin, and TNF-α, while upregulating E-cadherin expression. By blocking NF-κB/HIF-1α-mediated EMT and ECM remodeling, MTD effectively suppresses VM formation in ESCC under hypoxic tumor microenvironment conditions (Wang et al., 2025b).

In general, current studies on NPs and TCM formulas targeting VM formation in ESCC primarily achieve their effects by specifically targeting EphA2, blocking the EMT process, improving the hypoxic tumor microenvironment, and mediating ECM remodeling. However, current studies are relatively limited and primarily focus on TCM formulas. Future research should further clarify the molecular mechanisms by which individual compounds act on ESCC.

### 3.12 Regulatory mechanisms of cutaneous melanoma

Lycorine Hydrochloride (LH), the main bioactive alkaloid component of the medicinal plant Lycoris radiata, specifically inhibits VM formation in cutaneous melanoma (CM) C8161 cells by dose-dependently suppressing the activity of the VE-cadherin promoter (targeting the TATA/CACCC box region from −493 to +127 bp). This leads to downregulation of VE-cadherin transcription and protein expression, reduces its exposure on the cell membrane surface, and blocks intercellular connections among tumor cells (Liu et al., 2012b).

Nicotinamide (NA), the amide form of vitamin B3, is widely found in foods derived from both animals, plants, and microorganisms. Studies have shown that NA targets and inhibits VM in CM through epigenetic reprogramming. Specifically, NA dose-dependently downregulates the transcription and protein expression of VE-cadherin, directly disrupting the key molecular scaffold involved in VM channel formation. At the same time, it suppresses gene clusters related to vascular development and angiogenesis, and this effect is independent of the VEGF pathway (with mRNA levels decreased but no change at the protein level). Notably, the inhibitory effect of NA on VM is persistent, lasting for up to 1 month after drug withdrawal (Itzhaki et al., 2013).

Jatrorrhizine hydrochloride (JH), a protoberberine type alkaloid extracted from the dried rhizomes of Coptis chinensis, inhibits VM progression in CM by specifically downregulating VE-cadherin expression. This occurs without affecting the expression of other VM-related genes such as EPHB2, SFRP1, ZFP106, ROBO4, and WIPF2 (Liu et al., 2013).

Spondias cytherea Fruit Extract (SpE) is a plant-derived natural active compound. Studies have shown that SpE dose-dependently downregulates the expression of the CSCs marker CD133 and inhibits its functional activity, while significantly reducing the protein levels of NF-κB and COX-2, upregulating E-cadherin, and downregulating Vimentin expression. These effects markedly decrease the number of PAS-positive VM tubular structures, thereby further inhibiting VM in B16-F10 CM cells (Yolande et al., 2018).

Lupeol is a triterpenoid natural product found in various fruits and medicinal plants. In the B16-F10 CM cell model, Lupeol significantly downregulates the expression of the CSCs marker CD133, directly impairing the ability of CSCs to form VM tubular structures. Meanwhile, Lupeol downregulates VE-cadherin expression, hindering the maturation of bone marrow-derived endothelial progenitor cells (EPCs) and disrupting the synergistic interaction between angiogenesis and VM in the tumor microenvironment. Ultimately, Lupeol effectively blocks VM-mediated tumor microcirculation by inhibiting CSC stemness maintenance and EPC functional maturation (Bhattacharyya et al., 2019).

Piper capense Fruit Extract (PCFE) is a mixture rich in alkaloids, polyphenols, saponins, and other secondary metabolites, extracted from the African traditional medicinal plant Piper capense. Studies have shown that PCFE dose-dependently downregulates the expression of Vimentin and CD133, while upregulating E-cadherin expression to reverse the EMT process. It significantly reduces the number of CD31^-^/PAS^+^ VM tubular structures and can synergize with dacarbazine to overcome chemoresistance, jointly inhibiting VM formation in CM (Wamba et al., 2021).

Previous studies have reported that individual NPs can promote tumor VM formation. Other studies have shown that mixtures of natural compounds can also exert a promotive effect on tumor VM formation. Chicken embryo extract (CEE), a mixture rich in various natural bioactive components extracted from Gallus gallus, activates EMT by downregulating E-cadherin protein expression and upregulating Vimentin expression, leading to the loss of intercellular adhesion. This results in the acquisition of a mesenchymal phenotype and stem cell-like characteristics (loss of CD90/CD95/CD271 antigens), thereby promoting VM formation in Mel Ibr CM cells (Suraeva et al., 2017).

Various NPs reported in existing studies primarily target the core expression of VE-cadherin, regulate the EMT process, and inhibit the expression and function of the CSCs marker CD133. Through these mechanisms, they effectively disrupt intercellular connections in CM cells, reverse the pro-VM phenotypic transition, weaken the tube-forming ability of CSCs, and interfere with the synergistic interaction between angiogenesis and VM in the tumor microenvironment, ultimately suppressing VM formation in CM. Notably, some NPs demonstrate a sustained inhibitory effect, while others possess the potential to overcome chemoresistance.

### 3.13 Regulatory mechanisms of uveal (choroidal) melanoma

Genistein, a soy isoflavone abundantly present in soybeans, dose-dependently downregulates the mRNA and protein expression levels of VE-cadherin, reduces the number of CD34^-^/PAS^+^ channels *in vivo*, and inhibits the formation of VM structures in uveal melanoma (UM) (Cong et al., 2009).

In addition, studies have shown that luteolin can dose-dependently downregulate the phosphorylation levels of PI3K and Akt, thereby inhibiting the secretion of downstream VEGF and suppressing the formation of VM structures in C918 cells. By inhibiting the proliferation, migration, and invasion of UM cells, luteolin further disrupts VM formation, demonstrating a synergistic inhibitory effect on VM through targeting the PI3K/Akt signaling pathway and VEGF (Chen et al., 2022).

In choroidal melanoma (ChM), curcumin inhibits VM by downregulating the expression of EphA2 and blocking its downstream signaling, thereby suppressing the expression of PI3K, MMP2, and MMP9. This impairs the tumor cells’ ability to remodel the ECM, highlighting the role of curcumin in targeting the EphA2/PI3K/MMPs signaling pathway to inhibit VM formation in ChM (Chen et al., 2011).

Under hypoxic conditions, the activation of HIF-1α upregulates the expression of VEGF and PDGF, which synergistically promote the migration, invasion, and formation of VM tubular structures in ChM cells (Ma et al., 2024). Artesunate (ART), a sesquiterpene lactone derived from Artemisia annua, has been found to exert anti-VM effects in ChM by targeting and inhibiting the expression of HIF-1α protein, thereby downregulating VEGF and PDGF expression (Ma et al., 2024). Further studies have shown that ART significantly downregulates Wnt5a expression and inhibits the phosphorylation of Ca^2+^/calmodulin-dependent protein kinase II (CaMKII), promoting the degradation of HIF-1α and suppressing the expression of VE-cadherin, EphA2, VEGFA, VEGFR2, and PDGFR, thereby further inhibiting VM formation in ChM cells (Geng et al., 2021).

In general, NPs primarily exert their anti-VM effects by targeting the expression and activity of key VM molecules such as VE-cadherin, EphA2, and HIF-1α, and by inhibiting the activation of pro-VM signaling pathways including PI3K/Akt, Wnt/CaMKII, and downstream effectors of HIF-1α. These actions effectively disrupt tumor cell remodeling of the ECM, impede the expression of pro-angiogenic factors under hypoxic conditions, block signal transduction mediated by critical VM receptors, and inhibit cell migration and invasion, ultimately suppressing VM formation in uveal (choroidal) melanoma. Furthermore, certain NPs such as ART demonstrate the advantage of multi-pathway synergistic intervention, exerting regulatory effects across multiple pathways including hypoxia-related pathways, the VEGF/PDGF signaling axis, and calmodulin-mediated pathways. Notably, the pronounced hypoxia in the unique intraocular microenvironment of uveal melanoma-characterized by high vascularization and limited lymphatic drainage-may exacerbate its reliance on VM (Jager et al., 2020; Coupland et al., 2013). This could render VM a critical therapeutic target worthy of further exploration.

### 3.14 Regulatory mechanisms of renal cell carcinoma, nasopharyngeal carcinoma, and gallbladder carcinoma

In renal cell carcinoma (RCC), curcumin downregulates the expression of ETS-1 in a dose- and time-dependent manner. ETS-1 can directly bind to the promoter regions of VE-cadherin and MMP9, thereby suppressing the transcription and protein expression of these downstream effector molecules. Additionally, curcumin reduces the number of CD31^-^/PAS^+^ channels in RCC xenograft models. Collectively, these findings indicate that curcumin inhibits VM in RCC by targeting the ETS-1/VE-cadherin/MMP9 signaling pathway (Chong et al., 2024).

In nasopharyngeal carcinoma (NPC), studies have shown that Ginsenoside Rg3 concentration-dependently downregulates VEGF protein expression in HNE-1 cells and significantly inhibits the ability of NPC cells to form VM tubular structures *in vitro* (Wang et al., 2010).

Norcantharidin (NCTD) is a low-toxicity demethylated derivative of cantharidin, which is extracted from Mylabris phalerata. It dose-dependently downregulates the expression of PI3K, MMP-2, MT1-MMP, and Ln-5γ2, thereby blocking PI3K-mediated activation of MMPs. This inhibition suppresses the degradation of Ln-5γ2 and the release of its pro-migratory fragments γ2′ and γ2x, which are mediated by downstream MMP-2 and MT1-MMP (Zhang et al., 2014; Zhu et al., 2015). Consequently, NCTD promotes the disappearance of CD31^-^/PAS^+^ VM tubular structures *in vivo* in gallbladder carcinoma (GBC), disrupting the structure and function of VM and inhibiting GBC progression. It is important to note that Ln-5γ2 expression is downregulated in GBC but upregulated in osteosarcoma, which may cause some confusion for readers. This discrepancy mainly arises because, in osteosarcoma studies, RPS maintains the integrity of Ln-5γ2 by inhibiting MMPs, thereby reducing the production of Ln5γ2'/Ln5γ2x fragments that promote VM formation, ultimately disrupting the matrix microenvironment required for VM (Yao et al., 2022). In contrast, in GBC studies, NCTD blocks the overall expression level of Ln-5γ2, including both the intact protein and its cleaved fragments, thereby exerting a significant anti-VM effect by directly reducing the production of precursor fragments that promote VM (Zhang et al., 2014).

Current research on the biological and molecular mechanisms by which NPs inhibit VM in RCC, NPC, and GBC is relatively limited. Existing studies mainly focus on transcription factors that regulate CAMs expression and ECM remodeling in RCC, the interplay between VM and classical angiogenesis mediated by VEGFR in NPC, and ECM remodeling in GBC. Future investigations are needed to further elucidate the molecular mechanisms of various NPs targeting VM formation in these three cancers, thereby facilitating the development of effective VM-targeted therapies and advancing the translation of NPs from mechanistic research to clinical anti-VM applications.

## 4 Discussion

As an alternative blood supply mode of tumor microcirculation, VM has been widely recognized in recent years as a critical driver of invasion, metastasis, and therapeutic resistance in highly malignant tumors (Zhang et al., 2016). Compared to traditional endothelial cell-dependent angiogenesis, VM formation does not rely on vascular endothelial cells. Instead, it involves the spontaneous remodeling of tumor cells with high plasticity to form vessel-like channels (Luo et al., 2020). The formation mechanism is complex and involves multiple aspects, including the expression of CAMs, ECM remodeling, EMT, maintenance of tumor stemness, and reprogramming of the tumor microenvironment (Wei et al., 2021). Against this backdrop, therapeutic strategies targeting VM offer the unique advantage of disrupting non-endothelial blood supply pathways in tumors. This approach effectively overcomes the bottleneck of drug resistance encountered in conventional therapies and compensates for the alternative vascular mechanisms that tumors often develop following VEGF inhibition (Haiaty et al., 2021; Wamba et al., 2021). Furthermore, VM-targeted strategies exhibit the potential to effectively suppress more aggressive tumor subtypes (Cai et al., 2024; Zhu et al., 2020; Chiablaem et al., 2014; Xu et al., 2019). Notably, NPs and TCM exhibit distinct advantages in targeting VM, including multi-target and multi-pathway actions as well as low toxicity (Xiao et al., 2023; Cai et al., 2024; Yu et al., 2018). In addition, they have the capacity to remodel the tumor microenvironment and modulate immune responses, highlighting their promising potential for clinical application (Zhao et al., 2025; Zang et al., 2017). These benefits not only offer novel strategies to overcome current challenges in anti-tumor therapy but also lay a solid foundation for further research and clinical translation of NPs and TCM in the field of VM inhibition.

This study systematically reviews the molecular mechanisms by which NPs and TCM formulations inhibit VM formation across various tumor types, and summarizes multiple core intervention targets, including VE-cadherin, EphA2, MMPs, Twist1, HIF-1α, and PI3K/Akt. Studies have found that many NPs, such as curcumin, myricetin, luteolin, baicalein, and ginsenoside Rg3, can simultaneously target multiple key pathways. They inhibit the ability of tumor cells to form VM networks by blocking the EMT process, disrupting the cytoskeleton, suppressing ECM degradation, inducing ferroptosis, and regulating the expression of exosomal microRNAs (Chiablaem et al., 2014; Wang et al., 2022a; Zang et al., 2017; Zhang et al., 2020; Guo et al., 2014). In addition, some TCM formulations, such as YFJDT, Biejiajian pill, and CTC, have also demonstrated multi-component synergistic effects, reflecting the therapeutic advantages of combining holistic intervention with localized targeting in TCM (Wang et al., 2025c; An et al., 2018; Gao et al., 2024). Although several studies have reported that NPs and TCM inhibit tumor initiation and progression by inducing ferroptosis, investigations into how ferroptosis modulation affects VM remain scarce, highlighting the need for more in-depth research in this area (Hu et al., 2025; Tang et al., 2024).

Notably, the formation of VM is closely intertwined with EMT, CSCs, and the hypoxia-inducible factor HIF-1α through a highly interconnected signaling network. Multiple studies have demonstrated that EMT not only endows tumor cells with migratory and invasive capabilities but also promotes their transformation into VM-forming cells with endothelial-like characteristics (Wang et al., 2017; Zhang et al., 2021b; Li and Zhou, 2019). Moreover, CSCs serve as the “seeds” in VM formation due to their high plasticity and self-renewal capacity, with their stemness maintenance and differentiation largely dependent on the activation of signaling pathways such as HIF-1α, PI3K/Akt, Notch, and Wnt (Li et al., 2025; Lv et al., 2019; Karami Fath et al., 2022; Ordaz-Ramos et al., 2023). These signaling pathways are also the primary targets of many NPs and TCM, providing a systematic explanation for their anti-VM mechanisms and supporting their broad-spectrum antitumor properties.

Although numerous *in vitro* and animal studies have supported the efficacy of NPs and TCM formulations in inhibiting VM, many challenges remain in their clinical translation. First, the vast majority of studies focus on *in vitro* cell models and tumor xenograft mouse experiments, lacking support from large-scale clinical data. Most studies only verify the reduction of VM structures through CD31^-^/PAS^+^ or CD34^-^/PAS^+^ double staining, without systematically evaluating the impact of NPs and TCM on patient prognosis. Secondly, pharmacokinetic and pharmacodynamic studies on the inhibition of VM by NPs and TCM are insufficient, with their *in vivo* metabolic stability and dose optimization yet to be clearly defined. This uncertainty poses challenges in balancing efficacy and toxicity during clinical application. Additionally, significant differences exist among various NPs and TCM in terms of chemical properties, target specificity, and toxicity profiles, and there is currently a lack of unified screening and evaluation standards. Meanwhile, the therapeutic heterogeneity of NPs and TCM in different tumor microenvironments requires further investigation to identify the most suitable patient populations and tumor types for their application. The complexity of TCM formulations also poses challenges for mechanistic studies, as the material basis and target networks underlying their multi-component synergistic effects remain incompletely understood, making standardization and quality control of these formulations difficult.

Based on the above findings, to further advance the application of NPs and TCM formulations in VM therapy, future research urgently needs to focus on the following aspects: First, it is necessary to further explore under-investigated signaling nodes and regulatory networks involved in VM initiation and progression, aiming to identify core molecular targets with strong specificity and well-defined upstream and downstream mechanisms. Second, leveraging multi-omics data integration-including transcriptomics, proteomics, and metabolomics-combined with artificial intelligence-assisted analysis, systematic studies on the “efficacy–target–pathway” triad of NPs and TCM formulations should be conducted to comprehensively elucidate their mechanisms in VM intervention. Third, considering the poor solubility and low bioavailability of most NPs, optimization of drug delivery systems and formulation technologies is needed to enhance their bioavailability and tissue-targeting capabilities. Fourth, strengthening the investigation of synergistic effects between NPs, TCM formulations, and existing anti-angiogenic drugs could provide new combination strategies for anti-VM therapy. Fifth, efforts should be made to bridge the gap between preclinical research and clinical application by promoting representative NPs into early-phase (Phase I/II) clinical trials, thereby accelerating their translation into practical antitumor treatments. Only by establishing effective linkage mechanisms among basic research, technological approaches, and clinical practice can continuous optimization and breakthroughs in VM inhibition strategies be achieved.

## 5 Conclusion

In summary, NPs and TCM exhibit substantial potential in inhibiting tumor VM by disrupting non-endothelial-dependent tumor blood supply pathways through multi-target interventions. By targeting key mechanisms such as EMT, ECM remodeling, CSC maintenance, and hypoxia signaling, NPs and TCM can overcome the resistance limitations of conventional anti-angiogenic therapies, enhance antitumor efficacy, and improve prognosis in patients with aggressive tumors. Although significant progress has been achieved, knowledge gaps persist, including an incomplete understanding of VM regulatory networks, the pharmacokinetic and pharmacodynamic profiles of NPs and TCM, and therapeutic heterogeneity within tumor microenvironments. Challenges in clinical translation, such as data insufficiency, formulation standardization, and bioavailability optimization, must be prioritized to ensure safe application. To bridge these gaps, future research should prioritize: (1) identifying novel signaling nodes and integrating multi-omics combined with analyses of “drug-target-pathway” interactions; (2) optimizing drug delivery systems to enhance solubility and targeting; (3) exploring synergies with anti-angiogenic agents; (4) advancing Phase I/II clinical trials to accelerate translational validation. Ultimately, addressing these challenges could revolutionize VM-targeted therapies, incorporating NPs and TCM into precision oncology, markedly reducing tumor invasion, metastasis, and drug resistance, and promoting personalized and durable treatment strategies.
